# Cell Cycle Regulates Nuclear Stability of AID and Determines the Cellular Response to AID

**DOI:** 10.1371/journal.pgen.1005411

**Published:** 2015-09-10

**Authors:** Quy Le, Nancy Maizels

**Affiliations:** 1 Molecular and Cellular Biology Graduate Program, University of Washington School of Medicine, Seattle, Washington, United States of America; 2 Departments of Immunology, Biochemistry and Pathology, University of Washington School of Medicine, Seattle, Washington, United States of America; CABIMER, Universidad de Sevilla, SPAIN

## Abstract

AID (Activation Induced Deaminase) deaminates cytosines in DNA to initiate immunoglobulin gene diversification and to reprogram CpG methylation in early development. AID is potentially highly mutagenic, and it causes genomic instability evident as translocations in B cell malignancies. Here we show that AID is cell cycle regulated. By high content screening microscopy, we demonstrate that AID undergoes nuclear degradation more slowly in G1 phase than in S or G2-M phase, and that mutations that affect regulatory phosphorylation or catalytic activity can alter AID stability and abundance. We directly test the role of cell cycle regulation by fusing AID to tags that destabilize nuclear protein outside of G1 or S-G2/M phases. We show that enforced nuclear localization of AID in G1 phase accelerates somatic hypermutation and class switch recombination, and is well-tolerated; while nuclear AID compromises viability in S-G2/M phase cells. We identify AID derivatives that accelerate somatic hypermutation with minimal impact on viability, which will be useful tools for engineering genes and proteins by iterative mutagenesis and selection. Our results further suggest that use of cell cycle tags to regulate nuclear stability may be generally applicable to studying DNA repair and to engineering the genome.

## Author Summary

AID (Activation Induced Deaminase) deaminates cytosines in DNA to initiate immunoglobulin gene diversification and to reprogram the genome in early development. AID is potentially highly mutagenic, as it deaminates C to U within single-stranded regions. Here we show that AID abundance is regulated by cell cycle, and that high levels of nuclear AID are tolerated only in G1 phase. These results identify an unanticipated role for spatiotemporal regulation in balancing demands of AID-initiated mutagenesis and its potentially pathological outcomes.

## Introduction

Activation-induced cytosine deaminase (AID) initiates immunoglobulin (Ig) gene diversification in activated B cells by deaminating C to U [1,2]. Either UNG2 or MSH2/6 recognize and process this damage, and repair via error-prone pathways results in somatic hypermutation (SHM), class switch recombination (CSR) or gene conversion. AID-initiated damage can have pathological outcomes, evident as the chromosomal translocations associated B cell malignancies [3–7]. AID also participates in erasing CpG methylation to reprogram the genome in early development [8–12], promotes B cell tolerance [13,14], and limits autoimmunity [15,16].

AID is tightly regulated. AID localizes predominately to the cytoplasm but requires access to the nucleus to function. Posttranslational modifications and interactions with other proteins modulate cytoplasmic retention and nuclear import [17–19]. AID persistence in the nucleus is limited by proteosomal degradation [20,21] and by CRM1-dependent nuclear export [22–24]. Catalytic activity of AID can be improved by active site point mutations, but while those mutations accelerate Ig gene diversification they also stimulate translocation and compromise cell viability [25]. Mutation or deletion of the C-terminal region that includes the nuclear export signal (NES) can diminish AID stability and the efficiency of CSR, but compromises cell fitness [26,27].

AID abundance is constant during cell cycle [20,26], but several kinds of observations have suggested that cell cycle may regulate AID activity. In DT40 chicken B cells, brief treatment with leptomycin B (LMB), an inhibitor of CRM1-dependent nuclear export, increases nuclear AID signal in G1 phase cells [28]; Polη, which copies donor DNA in AID-initiated gene conversion, co-localizes with the diversifying Igλ_R_ allele predominately in G1 phase [29]; UNG2 removes uracils produced upon deamination by AID predominately in G1 phase [30]; and RPA initially accumulates at Ig switch regions in G1 phase [31].

We have now asked if cell cycle regulates subcellular localization, stability or physiological activity of AID. We demonstrate that nuclear degradation occurs more slowly in G1 phase than in S-G2/M phase cells. We show that mutations that affect regulatory phosphorylation or catalytic activity can alter AID stability and abundance. We directly test the role of cell cycle regulation by fusing AID to tags derived from cell cycle regulators CDT1 and Geminin [32] to destroy nuclear protein outside G1 or S-G2/M phase. We show that nuclear AID accelerates SHM and CSR, and is tolerated by cells in G1 phase, but compromises viability in S-G2/M phase. These results establish that cell cycle regulates abundance of nuclear AID and determines the ability of cells to respond to AID-initiated DNA damage. The AID derivatives that we have generated may be useful tools for engineering genes by iterative mutagenesis and selection, and cell cycle tags may be generally useful for studying DNA repair and recombination and RNA biogenesis, and for genome engineering.

## Results

### Nuclear AID is destabilized by ubiquitin-dependent proteolysis

**Fig 1:**
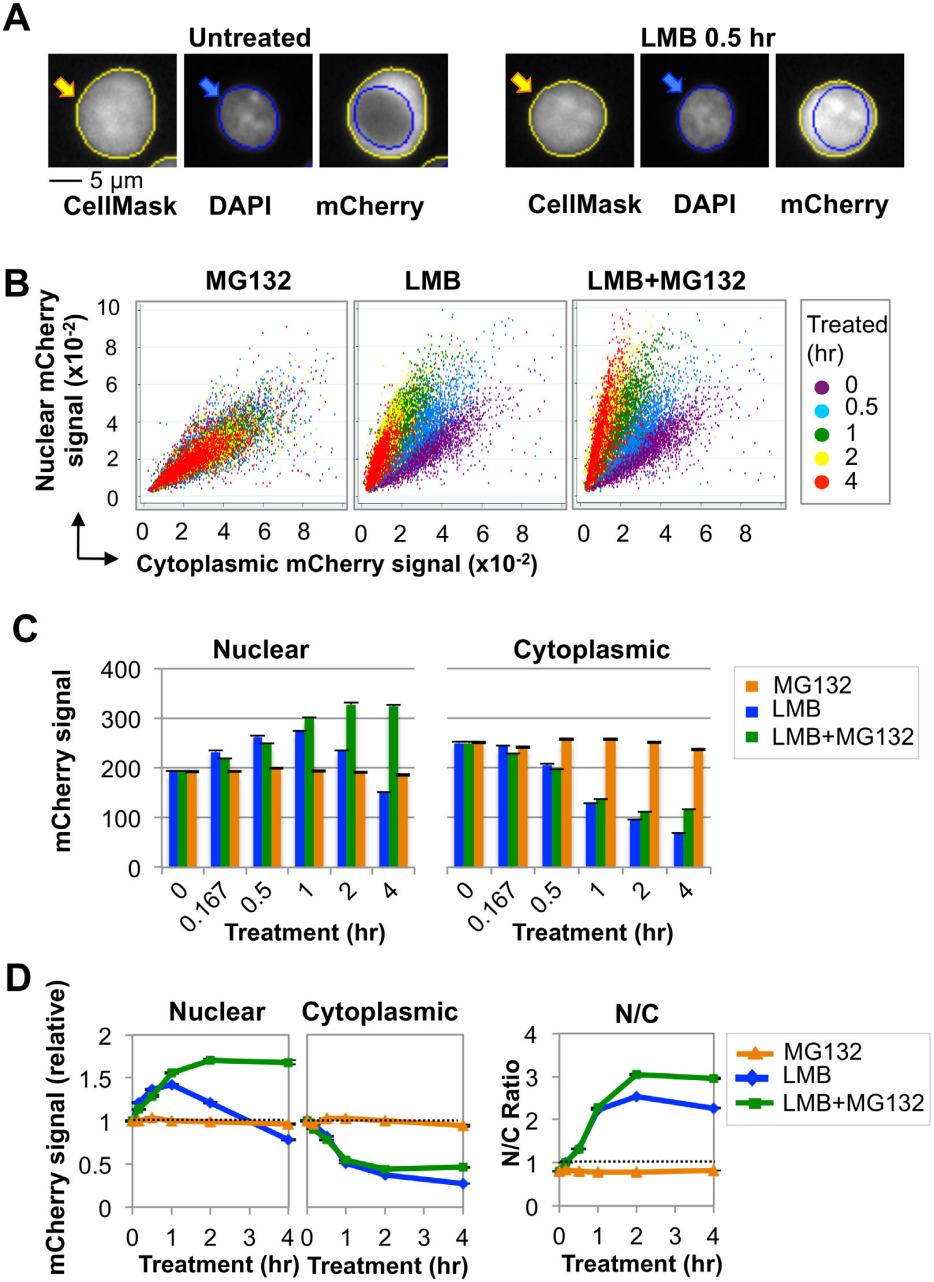
Nuclear AID is destabilized by ubiquitin-dependent proteolysis. (A) Representative examples of Ramos AID-mCherry transductants as analyzed by HCS, with whole cell boundary defined by HCS CellMask, yellow line; and nuclear boundary by DAPI, blue line. Typically, AID is cytoplasmic (N/C < 1), but treatment with LMB inhibits nuclear export of AID (N/C>1). In the examples shown, N/C = 0.80 (untreated) and 1.34 (0.5 hr LMB). (B) Scatter plots of nuclear vs. cytoplasmic mCherry (mCh) signals for untreated Ramos AID-mCherry transductants or for cells treated with MG132, LMB, or LMB+MG132 as indicated. (C) Quantification of total nuclear and cytoplasmic AID-mCherry signal by HCS, following treatment with MG132, LMB or LMB+Mg132 for indicated times. (D) Quantification of nuclear and cytoplasmic AID-mCherry signal and N/C ratio, relative to untreated cells, at indicated times post-treatment with MG132, LMB, or both. This analysis was repeated 3 more times for LMB treatment (see S3 Fig), and once for MG132 and LMB+MG132 treatment. Dotted line represents no change (fold change of 1). Each point represents a population average, and black bars (too small to be discerned readily) represent SEM of the population.

We analyzed subcellular distribution of AID in the human B cell line, Ramos, transduced with a lentiviral construct expressing human AID fused at the C-terminus to the mCherry fluorescent protein (AID-mCherry). Ramos B cells express endogenous AID and actively hypermutate their Ig genes, so the pathways that regulate and respond to damage by AID are intact; and both endogenous and exogenous protein will contribute to total AID abundance. Cells were analyzed by high content screening (HCS) microscopy [33], a flow-based approach that automatically quantifies signals per unit area (pixels) in each compartment of each cell (Fig 1A; see Materials and Methods). Control experiments verified that cell cycle was not perturbed significantly by up to 4 hr of culture with MG132, an inhibitor of the ubiquitin-dependent 26S proteasome; LMB; or MG132+LMB (S1 Fig). MG132 treatment had little effect on nuclear and cytoplasmic signals in populations of AID-mCherry transductants; while treatment with LMB or both LMB+MG132 rapidly increased nuclear signal in most cells (Fig 1B). Quantification established that nuclear signal was unaffected by MG132 treatment; rapidly increased (1.5-fold) and then declined in response to LMB treatment; and increased (1.7-fold) and plateaued in response to treatment with both LMB+MG132 (S1 Table and Fig 1C and 1D). Cytoplasmic signal was unaffected by MG132 treatment, but diminished upon treatment with LMB or LMB+MG132, paralleling the increase in nuclear signal (S1 Table and Fig 1C and 1D). Similarly, the ratio of nuclear to cytoplasmic AID (N/C) was unaffected by treatment with MG132 alone; and increased and plateaued in response to LMB or LMB+MG132 treatment (Fig 1D, right). These results are consistent with previous reports that AID undergoes nuclear proteolysis [20,21].

### Nuclear AID is more stable in G1 phase than in S or G2/M phases

**Fig 2:**
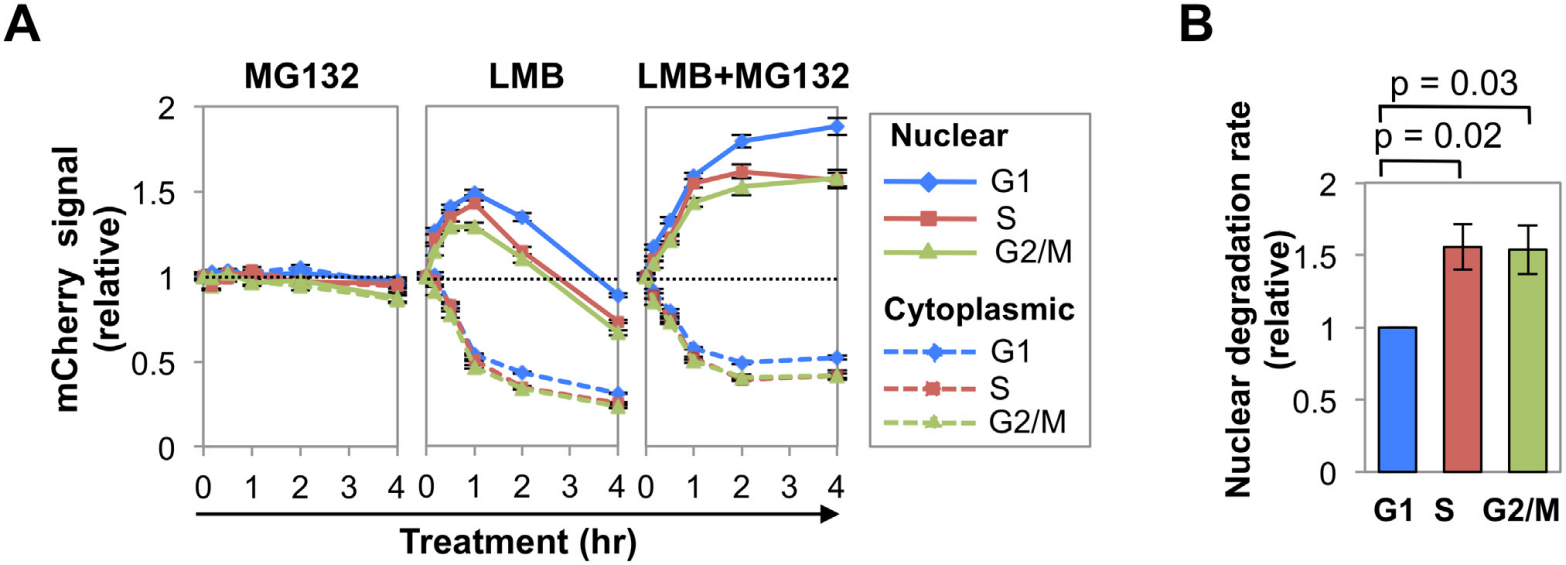
Nuclear AID is degraded more slowly in G1 phase than S-G2/M phases. (A) Representative analysis of kinetics of response of AID-mCherry nuclear (solid lines) and cytoplasmic (dashed lines) signals to treatment with MG132, LMB or LMB + MG132 in G1, S and G2/M phase cells. This analysis was repeated 3 more times for LMB treatment (see S3 Fig), and once for MG132 and LMB+MG132 treatment. Each point represents a population average, and black bars represent SEM of the population, which are too small to discern. Dotted line represents no change (fold change of 1). (B) Relative rates of nuclear degradation of AID-mCherry in LMB-treated cells in G1, S and G2/M phases. Rates were estimated as the slope of the line defined by the population averages at 1 and 2 hr of treatment, in 4 independent experiments (Figs 2A and S3). Values are presented relative to the slope in G1 phase. SEM, black bars. Significance (p values) shown above graph were determined by two-tailed, unpaired Student’s t-test, assuming unequal variances.

We used HCS to quantify AID-mCherry subcellular distribution in Ramos B cells in each phase of cell cycle, and the response to inhibitors of proteolysis and nuclear export (Fig 2). G1, S, and G2/M phase cells were distinguished by ranking DNA content as determined by total DAPI signal, and specific fractions of the population assigned to G1, S and G2/M phases (S2A Fig). HCS results were expressed in terms of average signal, to ensure independence of cell size, which increases during cell cycle (S2B Fig); and total and average mCherry signal both increased with DNA content (S2C, S2D, and S2E Fig).

Treatment with MG132 (Fig 2A) had little effect on nuclear or cytoplasmic AID-mCherry signals in any phase of cell cycle. Treatment with LMB or LMB+MG132 (Fig 2A) caused the cytoplasmic signal to drop by 50% in all stages of cell cycle, evidence of the importance of nuclear export in maintaining cytoplasmic signal. Treatment with LMB caused the nuclear AID-mCherry signal to increase (0–1 hr) and then drop, while treatment with LMB+MG132 caused this signal to increase and then plateau; thus the drop in nuclear signal following treatment with LMB alone was due to proteolysis. Notably, LMB treatment caused a sharper initial increase and more gradual decrease in nuclear signal in G1 phase than S or G2/M phase cells; while LMB+MG132 treatment resulted in a significantly higher relative signal in G1 phase than S or G2/M phase cells (at 2 hr, G1 vs. S, p = 1.4x10^-3^; G1 vs. G2/M, p = 1.8 x10^-5^; Fig 2A, right and S2 Table). Thus, nuclear stability of AID-mCherry is cell cycle dependent, and stability is highest in G1 phase.

Comparison of the slopes of the LMB response curves between the 1 and 2 hr time points (Fig 2A, center) suggested that degradation occurred more rapidly in S-G2/M phase than G1 phase. To quantify the difference, we calculated the average rate of loss of nuclear signal between 1–2 hr of treatment, as defined by the slope of the line between these time points, for 4 independent experiments (Figs 2A and S3). Rates of initial degradation were 1.56-fold and 1.54-fold higher in S and G2/M phases (p = 0.02 and 0.03, respectively; Fig 2B) than in G1 phase. We conclude that nuclear AID-mCherry is degraded more rapidly in S and G2/M phase than in G1 phase.

### AID phosphorylation and catalytic activity regulate nuclear abundance

AID undergoes phosphorylation on at least five different residues: Ser3, Thr27, Ser38, Thr140, Tyr184. To ask how abundance might be regulated in the course of cell cycle, we focused on two of them, Ser3 and S38, sites of negative and positive regulation, respectively. Phosphorylation of AID on Ser3 downregulates SHM and CSR, and protein phosphatase 2A (PP2A) removes this modification [34]. PP2A is a tumor suppressor that controls cell cycle progression by modulating phosphorylation of G1/S cyclins to maintain appropriate levels [35], making Ser3 modification of interest as a target of cell cycle regulation. Phosphorylation of AID on Ser38 by protein kinase A (PKA) promotes robust SHM and CSR, in part by enabling interaction with RPA [36–40], which is in turn cell cycle-regulated [41]. The fifth derivative analyzed was AID^H56A^-mCherry, mutant at a conserved histidine that is one of three residues that coordinates an active site zinc ion essential for deaminase activity [42].

**Fig 3:**
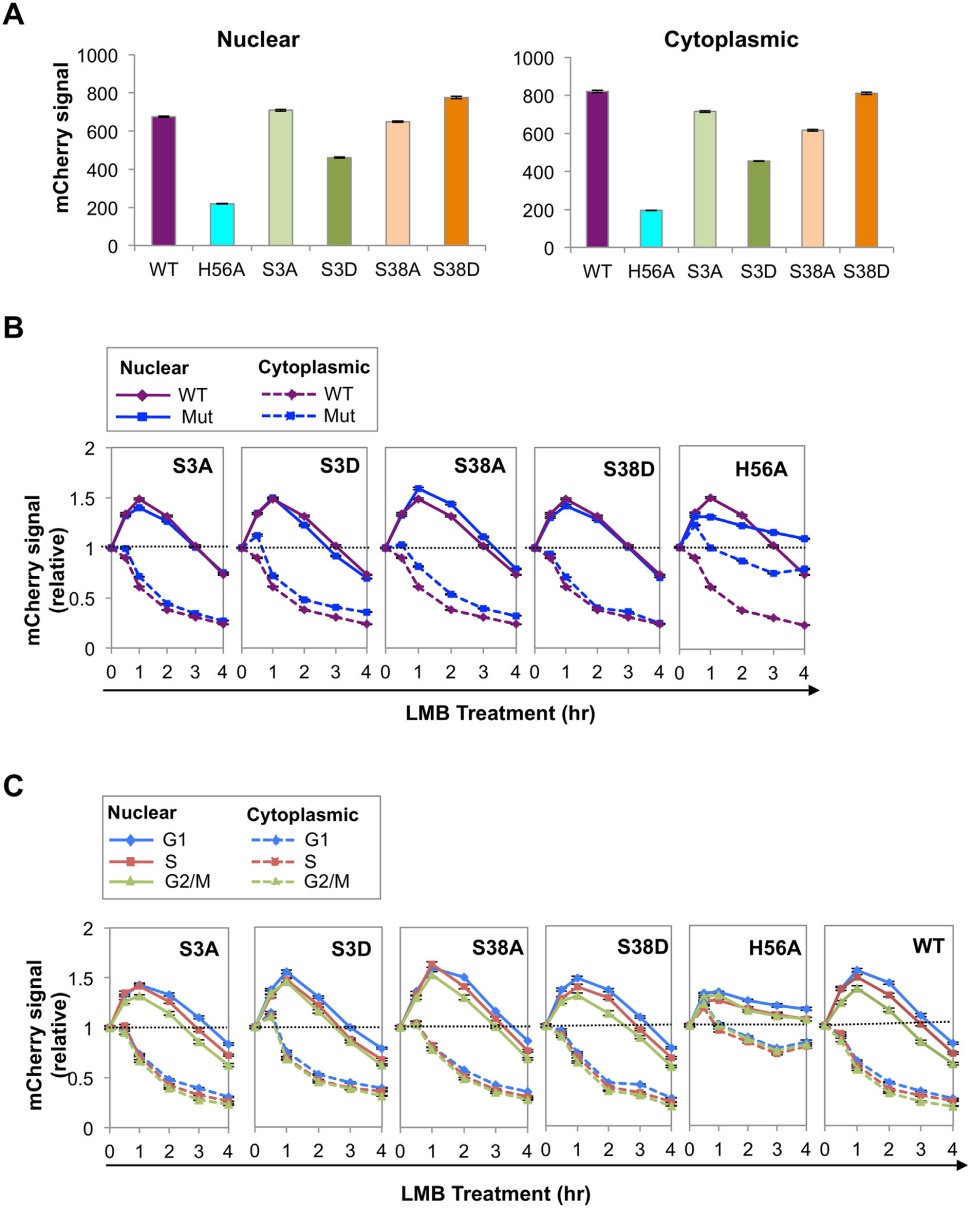
Phosphorylation and catalytic activity regulate AID abundance and nuclear stability. (A) Nuclear and cytoplasmic mCherry signals from indicated mutants, as determined by HCS analysis, expressed in arbitrary units. Population average signals are shown; black bars represent SEM of the population, which are too small to discern. (B) Representative HCS analysis of kinetics of response of nuclear (solid lines) and cytoplasmic (dashed lines) signals of indicated AID-mCherry mutants to treatment with LMB, relative to untreated cells. Each point represents a population average, and black bars represent SEM of the population, which are too small to discern. Dotted line represents no change (fold change of 1). (C) Representative HCS analysis of kinetics of response of nuclear (solid lines) and cytoplasmic (dashed lines) signals of indicated AID-mCherry mutants to treatment with LMB in G1, S and G2/M phase cells. Each point represents a population average, and black bars represent SEM of the population, which are too small to discern. Dotted line represents no change (fold change of 1). Analysis of WT AID was carried out in the same experiment and is shown at right for comparison.

We quantified mCherry signals in nuclei and cytoplasm of Ramos cells expressing WT AID-mCherry or AID point mutants (Fig 3A). The AID^S3A^-mCherry signal was comparable to that of AID-mCherry, while the signal of the corresponding phosphomimetic mutant, AID^S3D^-mCherry, was reduced to approximately 60–70% that of WT. The nuclear AID^S38A^-mCherry signal was slightly lower than that of AID-mCherry, while the signal of the corresponding phosphomimetic mutant, AID^S38D^-mCherry, was slightly higher. These results suggest that phosphorylation at Ser3 regulates AID abundance.

The nuclear and cytoplasmic signals of AID^H56A^-mCherry were much lower than those of WT AID-mCherry (25–30%; Fig 3A). While we cannot rule out the possibility that the reduced signal is due to perturbation of overall structure by the H56A mutation, mutation of His to Ala at the corresponding residue of the highly related active site of mouse adenosine deaminase has been shown to reduce catalytic activity but not affect secondary or tertiary structure [43]. Reduced abundance was not B cell specific, as mutation of His to Arg at this position (AID^H56R^-mCherry) also reduced abundance (S4A Fig). Furthermore, reduced abundance was not an artifact of cloning, as the signal of a reversion mutant (AID^H56A-H^) was comparable to that of the AID-mCherry control (S4B Fig).

To ask if differences in AID abundance reflected relative rates of degradation, we assayed nuclear and cytoplasmic signals of each mutant over the course of 4 hr treatment with LMB, and plotted signals in treated relative to untreated cells (Fig 3B). At 1–2 hr following LMB treatment, the nuclear degradation rate of AID^S3D^-mCherry was more rapid than that of AID^S3A^-mCherry or AID-mCherry, consistent with the lower abundance of AID^S3D^-mCherry. The nuclear degradation rates of AID^S38A^-mCherry and AID^S38D^-mCherry exhibited no clear differences (Fig 3B). The nuclear degradation rate of AID^H56A^-mCherry was apparently lower than that of WT AID-mCherry (Fig 3B). This was surprising in light of the low abundance of AID^H56A^-mCherry (Fig 3A), and may reflect difficulty in measuring small differences in relative signals of a low-abundance protein.

We asked if cell cycle affected degradation rates by comparing signals in G1, S and G2/M phase cells, untreated or treated with LMB (Figs 3C and S5). At 1–2 hr post-treatment with LMB, AID^S3D^-mCherry exhibited more rapid nuclear degradation in G1 phase than AID^S3A^-mCherry, consistent with negative regulation by phosphorylation at Ser3. AID^S38A^-mCherry and AID^S38D^-mCherry did not exhibit clear differences (Fig 3C). The rate of loss of the AID^H56A^-mCherry signal did not vary among G1, S and G2/M phases (Fig 3C). These results show that phosphorylation at Ser3 may contribute to cell cycle regulation of AID by increasing the rate of degradation in G1 phase.

### Cell cycle regulation conferred by CDT1 and GEM tags

**Fig 4:**
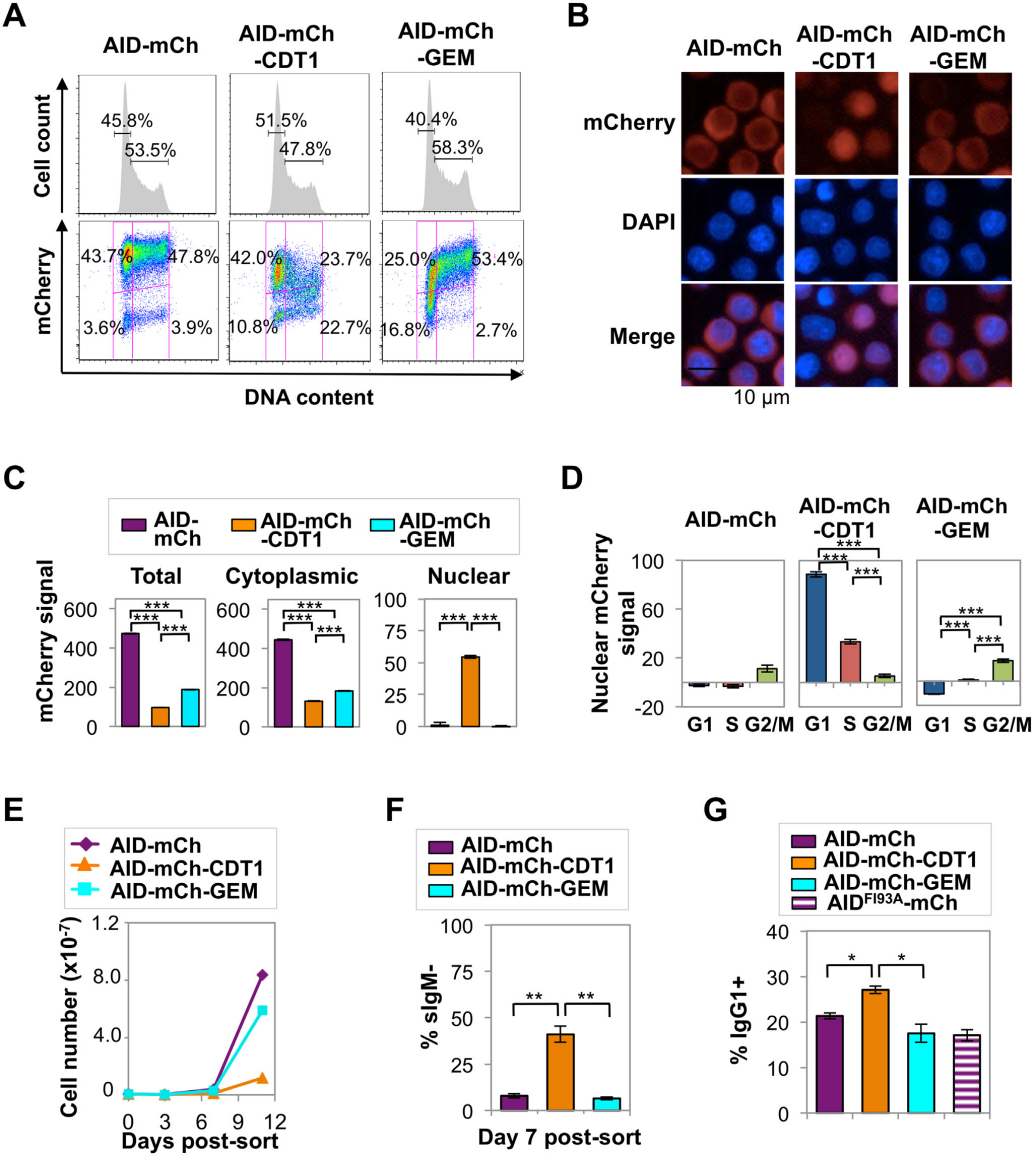
AID-mCherry CDT1 reduces viability and accelerates Ig gene diversification. (A) Flow cytometry of indicated Ramos transductants, showing cell number relative to DNA content and percent of cells in G1 or S-G2/M phases (above), and mCherry signal and fraction of population in each quadrant (below). (B) Representative fluorescence images of indicated transductants, showing mCherry, DAPI and merged signals. (C) Quantification of total, cytoplasmic and nuclear mCherry signals for indicated transductant populations as determined by HCS microscopy, showing the population average and SEM. ***, p<10^−10^ as determined by two-tailed, unpaired Student’s t-test, assuming unequal variances. (D) Nuclear mCherry (arbitrary units) signal in G1, S and G2/M phase cells in indicated transductant populations. Data presented and analyzed as in panel *c*. (E) Representative counts of viable cells for indicated transductants at days 3, 7, and 11 after sorting recent transductants for mCherry+. (F) Percentage of sIgM- cells at day 7 after sorting recent transductants for mCherry+ cells; average from 4 independent experiments. **, p < 0.005 as determined by two-tailed, unpaired Student’s t-test, assuming unequal variances. (G) Percentage of IgG1+ cells in cultures of indicated primary murine B cell transductants at day 4 of in vitro stimulation. *, p < 0.05 as determined by two-tailed, unpaired Student’s t-test, assuming unequal variances.

With the goal of restricting the presence of AID-mCherry in the nucleus to G1 or S/G2-M phases, we fused AID-mCherry to tags derived from the CDT1 and GEM cell cycle regulators, which target a fused protein for destruction in the nucleus in S-G2/M phase (CDT1) or G1/early S phase (GEM) [32]. Control experiments confirmed that, in Ramos B cells, these tags fused to monomeric Kusabira Orange 2 (mKO2) or monomeric Azami-Green (mAG) promoted nuclear localization (S6A Fig) and conferred the predicted cell cycle regulation: signals from mKO2-CDT1 or mAG-GEM were restricted to G1 phase or late G1/S-G2/M phase, respectively (S4B Fig). Expression of AID-mCherry-CDT1 or AID-mCherry-GEM did not disrupt the cell cycle profile of Ramos B cells (Fig 4A). However, regulation directed toward AID appeared to override some predicted effects of each tag. FACS analysis showed that restriction of the AID-mCherry-CDT1 signal to G1 phase was incomplete (Fig 4A), in contrast to G1 phase restriction evident for an analogous CDT1 tagged fluorescent protein not fused to AID, mKO2-CDT1 (S4B Fig). Furthermore, immunofluorescence microscopy identified no nuclear signal among cells expressing AID-mCherry-GEM (Fig 4B), in contrast to the strong nuclear signal among some (but not all) cells expressing AID-mCherry-CDT1 (Fig 4B) or mAG-GEM (S6B Fig). Nonetheless both the CDT1 and GEM tags did target the fusion protein for nuclear degradation during a portion of cell cycle, as predicted, as HCS analysis showed that total and cytoplasmic mCherry signals were significantly lower in AID-mCherry-CDT1 and AID-mCherry-GEM relative to AID-mCherry transductant populations (p = 0; S3 Table and Fig 4C). Active nuclear export was confirmed by showing that treatment with LMB or LMB+MG132 caused a comparable increase in nuclear signal (relative to untreated cells) in AID-mCherry, AID-mCherry-CDT1 and AID-mCherry-GEM transductants (S7 Fig).

The nuclear localization of AID-mCherry-CDT1 could reflect more rapid nuclear import. However, while the nuclear signal and the ratio of nuclear to cytoplasmic signal (N/C) peaked more quickly in AID-mCherry-CDT1 than in AID-mCherry or AID-mCherry-GEM transductants following treatment with LMB (S7 Fig), this modest increase does not fully explain the strong nuclear signal in a significant fraction of AID-mCherry-CDT1 transductants. In addition, HCS analysis showed that while the AID-mCherry-CDT1 nuclear signal was greatest in G1 phase cells, it was also evident in S phase cells. This suggested that AID-mCherry-CDT1 exported from the nucleus in G1 phase may re-enter in S phase to create a signal before it is targeted for proteolysis by the CDT1 tag. This possibility is addressed experimentally below.

HCS analysis also showed that AID-mCherry and AID-mCherry-GEM signals were exclusively cytoplasmic, independent of cell cycle (S4 Table and Fig 4D). Combined with the evidence that AID is degraded more rapidly in S and G2-M phases than in G1 phase (Fig 3), the absence of nuclear signal in AID-mCherry-GEM transductants suggests that mechanisms targeted to the GEM tag may promote its nuclear proteolysis in G1 phase, while mechanisms targeted to AID promote its proteolysis or nuclear export in other stages of cell cycle.

### AID-mCherry-CDT1 expression reduced viability and accelerated Ig gene diversification

The distinctive spatiotemporal regulation of AID-mCherry, AID-mCherry-CDT1 and AID-mCherry-GEM allowed us to analyze the physiological consequences of nuclear AID at different stages of cell cycle. Strikingly, AID-mCherry-CDT1 transductants exhibited diminished cell viability relative to AID-mCherry or AID-mCherry-GEM transductants (Fig 4E). This suggested that nuclear AID can compromise fitness.

sIgM loss frequency was 7.9% in AID-mCherry transductants, 41.1% (p = 0.003) in AID-mCherry-CDT1 transductants, and 6.5% in AID-mCherry-GEM transductants, in all cases above frequencies in transductants expressing the AIDH56A-mCherry catalytic mutant or mock transductants (Figs 4F and S8). Similar results were obtained in assays of Ramos AID-mKO2-CDT1 and AID-mKO2-GEM transductants, which carry an mKO2 fluorescent tag which is degraded more rapidly than the mCherry tag (S8 Fig). Thus, the CDT1 tag accelerated AID-initiated SHM in Ramos B cells.

We assayed the effects of the tagged AID derivatives on CSR by transducing primary murine B cells with AID-mCherry, AID-mCherry-CDT1 or AID-mCherry-GEM retroviral expression constructs, and culturing cells in vitro with IL-4 and anti-CD40 to stimulate CSR. The mCherry signal in transduced primary B cells proved to be barely 10-fold higher than the background for FACS analysis (S9A Fig). This was not sufficiently high to permit HCS analysis, so instead we assayed the physiological outcome of expression of the tagged proteins. Among AID-mCherry-CDT1 transductants of primary stimulated B cells, a significantly greater average fraction of cells switched to IgG1+ (27%) than among AID-mCherry transductants (21%; p = 0.006) or AID-mCherry-GEM (18%; p = 0.026) transductants (Figs 4G and S9B). Thus, AID-mCherry-CDT1 expression accelerated Ig gene diversification, as evident by both an increased frequency of SHM in Ramos transductants and an increased frequency of CSR in primary B cell transductants.

**Fig 5:**
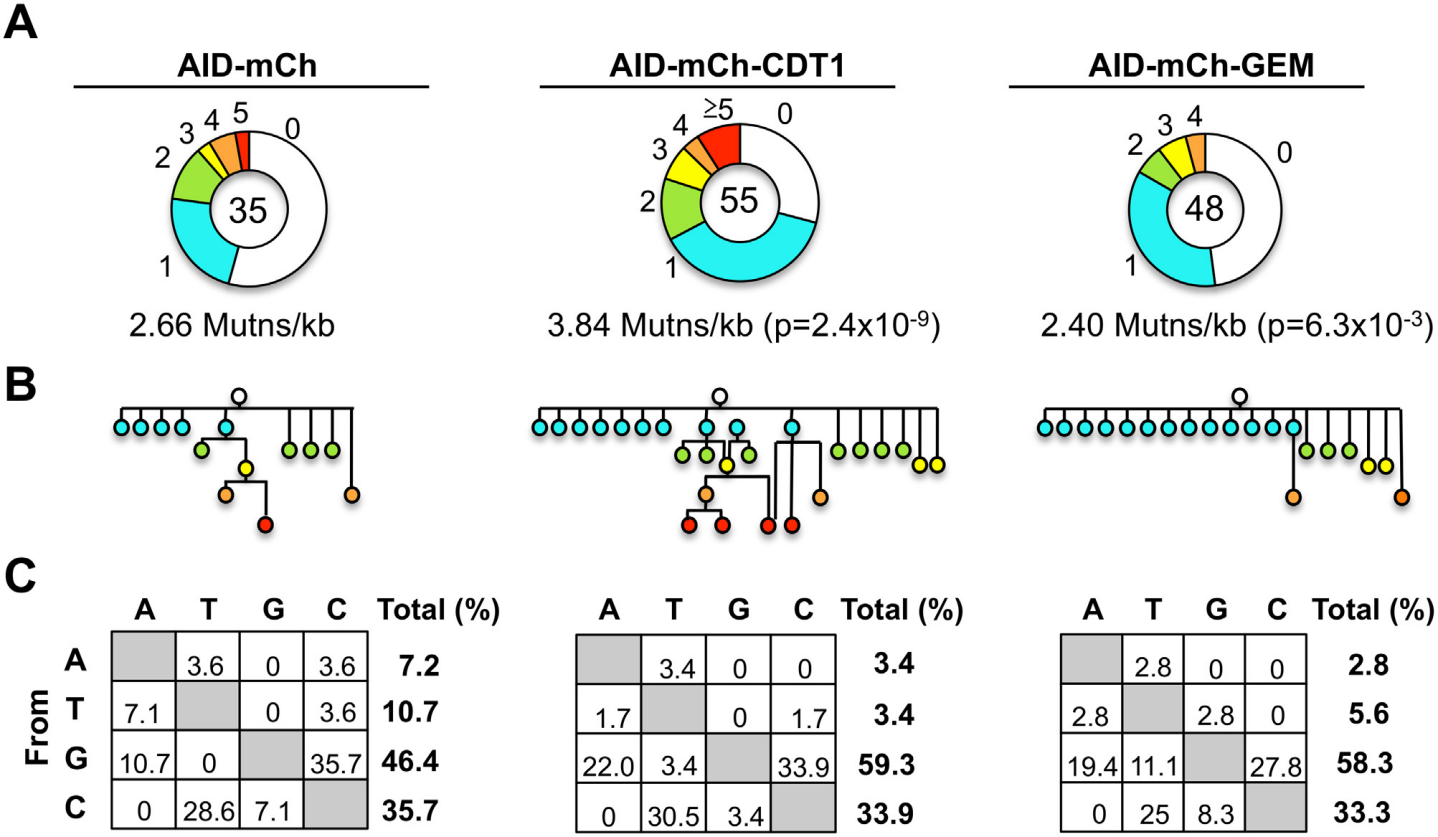
Frequencies and spectra of mutations at rearranged IgV_H_ regions. (A) Pie charts of hypermutation per IgV_H_ region for indicated Ramos B cell transductants, showing numbers of sequences analyzed (center) and proportions sequences exhibiting 0,1, 2, 3, 4, ≥5 mutations. Statistical significance determined by χ^2^ test using data from AID-mCherry transductants as expected values. (B) Genealogies of mutants in transductant populations, based on sequences of V_H_ regions (S10 Fig) including only sequences with distinct mutation spectra. Circles indicate total numbers of point mutations, color-coded as above. (C) Mutation spectra of indicated transductants, showing percentage of each possible single nucleotide substitution among all point mutations, with percentage of all point mutations that occur at each nucleotide shown on the right.

To determine frequencies and spectra of SHM, we sequenced IgV_H_ regions amplified from single Ramos B cells (S10 Fig). AID-mCherry-CDT1 transductants accumulated more mutations and more mutations per V region than AID-mCherry transductants (p = 2.4x10^-9^; Fig 5A). Point mutations at G or C accounted for over 80% of mutations in all transductants, accompanied by a few deletions and insertions (S11 Fig), similar to other analyses of SHM in Ramos B cells and derivatives expressing AID-GFP [44–46]. Accelerated SHM was further evident in diagrams of mutant lineages (Fig 5B). Even though the total numbers of sequences analyzed was relatively small, some interesting features are still worth noting. There were fewer mutations at A or T in AID-mCherry-CDT1 or AID-mCherry-GEM transductants relative to AID-mCherry transductants (6.8%, 8.4% and 17.9%, respectively; Fig 5C); and the fraction of transversion mutations from G to T was greater in AID-mCherry-GEM than AID-mCherry or AID-mCherry-CDT1 transductants (11.1%, 0% and 3.4% respectively; Fig 5C).

### Elevated nuclear AID is tolerated in G1 phase but not in S-G2/M phase cells

**Fig 6:**
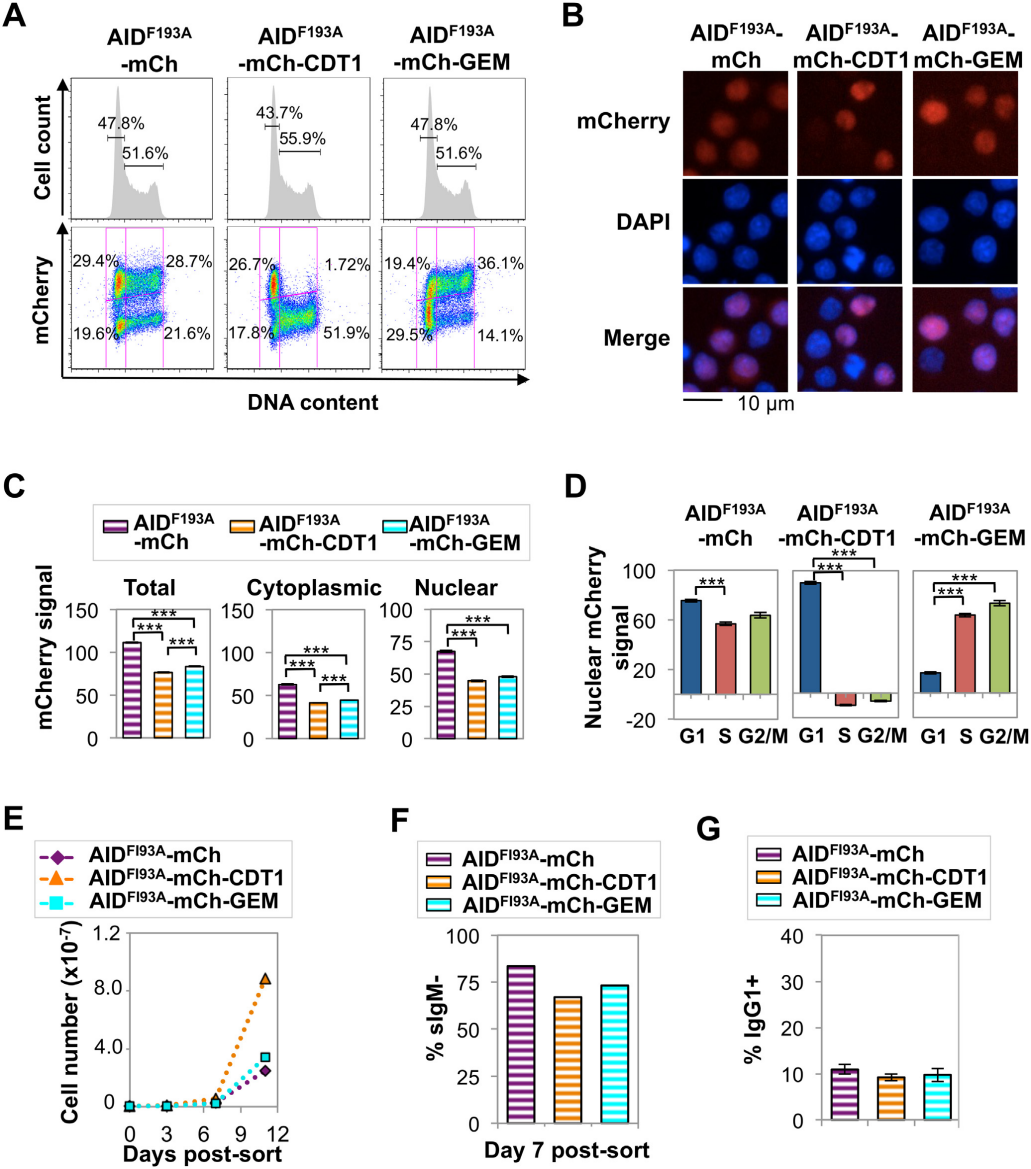
Elevated nuclear AID is tolerated in G1 phase but toxic in S-G2/M phase. (A) Flow cytometry of indicated Ramos transductants, showing cell number relative to DNA content and percent of cells in G1 or S-G2/M phases (above), and mCherry signal and fraction of population in each quadrant (below). (B) Representative fluorescence images of indicated transductants, showing mCherry, DAPI and merged signals. (C) Quantification of total, cytoplasmic and nuclear mCherry signals by HCS microscopy for indicated transductant populations, showing population average and SEM. Nuclear signals as determined by HCS were corrected for cytoplasmic baseline (see Materials and Methods). ***, p<10^−10^ as determined by two-tailed, unpaired Student’s t-test, assuming unequal variances. (D) Nuclear mCherry (arbitrary units) signal in G1, S and G2/M phase cells in indicated transductant populations. Population average and SEM of a representative experiment are shown. ***, p<10^−10^ as determined by two-tailed, unpaired Student’s t-test, assuming unequal variances. (E) Representative counts of viable cells for indicated transductants at days 3, 7, and 11 after sorting recent transductants for mCherry+ cells (see also S12 Fig). (F) Percentage of sIgM- cells at day 7 after sorting recent transductants for mCherry+ cells. (G) Percentage of IgG1+ cells in cultures of indicated primary murine B cell transductants at day 5 of in vitro stimulation. *, p < 0.05 as determined by two-tailed, unpaired Student’s t-test, assuming unequal variances.

A nuclear AID-mCherry-CDT1 signal was evident in both G1 and S phase cells (Fig 4D). As AID is actively exported from the nucleus, this signal was likely to derive from protein that had been exported in G1 phase and re-entered in S phase and escaped proteolysis long enough to produce a signal. To test this, we analyzed spatiotemporal localization of derivatives carrying the well-characterized AID^F193A^ mutation which prevents nuclear export, and also reduces protein abundance and accelerates SHM [26]. Flow cytometry showed that expression of AID^F193A^-mCherry, AID^F193A^-mCherry-CDT1 or AID^F193A^-mCherry-GEM did not disrupt the cell cycle profile in Ramos B cells (Fig 6A). Fluorescence microscopy identified clear nuclear signals in each transductant population, consistent with inhibition of nuclear export (Fig 6B). In the AID^F193A^-mCherry-CDT1 transductant population, essentially no S-G2/M phase cells exhibited mCherry signal, in contrast to AID-mCherry-CDT1 transductants (cf. Figs 4A, 4D, 6A and 6D and S4 Table). This establishes that nuclear export and re-entry is the source of the AID-mCherry-CDT1 nuclear signal in S phase cells.

HCS analysis showed that total and cytoplasmic mCherry signals were significantly lower in AID^F193A^-mCherry-CDT1 and AID^F193A^-mCherry-GEM transductants than in AID^F193A^-mCherry transductants, as predicted for tags that target the protein for nuclear degradation during a portion of cell cycle (S3 Table and Fig 6C). Comparison of AID-mCherry vs. AID^F193A^-mCherry and AID-mCherry-GEM vs. AID^F193^-mCherry-GEM transductants showed that the F193A mutation reduced total and cytoplasmic signals several-fold or more, and greatly increased nuclear signals; while signals were reduced to a lesser extent in AID-mCherry-CDT1 relative to AID^F193^-mCherry-CDT1 transductants (cf. Figs 4C and 6C and S3 Table).

HCS further showed persistent nuclear localization of AID^F193A^-mCherry and AID^F193A^-mCherry-GEM in all phases of cell cycle, while nuclear localization of AID^F193A^-mCherry-CDT1 occurred exclusively in G1 phase (S4 Table and Fig 6D). AID^F193A^-mCherry and AID^F193A^-mCherry-GEM transductants exhibited diminished cell viability, but AID^F193A^-mCherry-CDT1 transductants proliferated robustly in several independent analyses (Figs 6E and S12). We conclude that cells tolerate high levels of nuclear AID in G1 phase, but not at other stages of cell cycle.

The Ramos AID^F193A^-mCherry, AID^F193A^-mCherry-CDT1 and AID^F193A^-mCherry-GEM transductants all exhibited greatly elevated sIgM loss rates (Fig 6F), as previously documented for AID^F193A^ mutants [26]. CSR requires an intact AID C-terminal region [26,47], and consistent with this CSR to IgG1 was not accelerated in primary B cells expressing AID derivatives bearing the F193A mutation (Figs 6G and S9C). Notably, AID^F193A^-mCherry-CDT1 was distinguished by its ability to accelerate SHM without vastly compromising cell viability. This will make AID^F193A^-mCherry-CDT1 a useful tool for accelerating mutagenesis in platforms designed to optimize evolution of antibodies and other targets.

## Discussion

We have shown that cell cycle regulates AID nuclear stability, and we have confirmed the importance of G1 phase in Ig gene diversification, previously suggested by several kinds of evidence [28–31]. We have identified regulatory phosphorylation at Ser3 as one determinant of nuclear stability, and we have shown that catalytic activity is one determinant of AID abundance. The CDT1 and GEM tags that we have used to regulate AID nuclear stability may be useful for studying other proteins, and they may have practical applications in engineering genes and proteins. The importance of cell cycle in normal regulation of AID suggests that dysregulation of this control could promote AID-initiated mutagenesis in cancer.

### G1 phase is the sweet spot for AID-initiated mutagenesis

**Fig 7:**
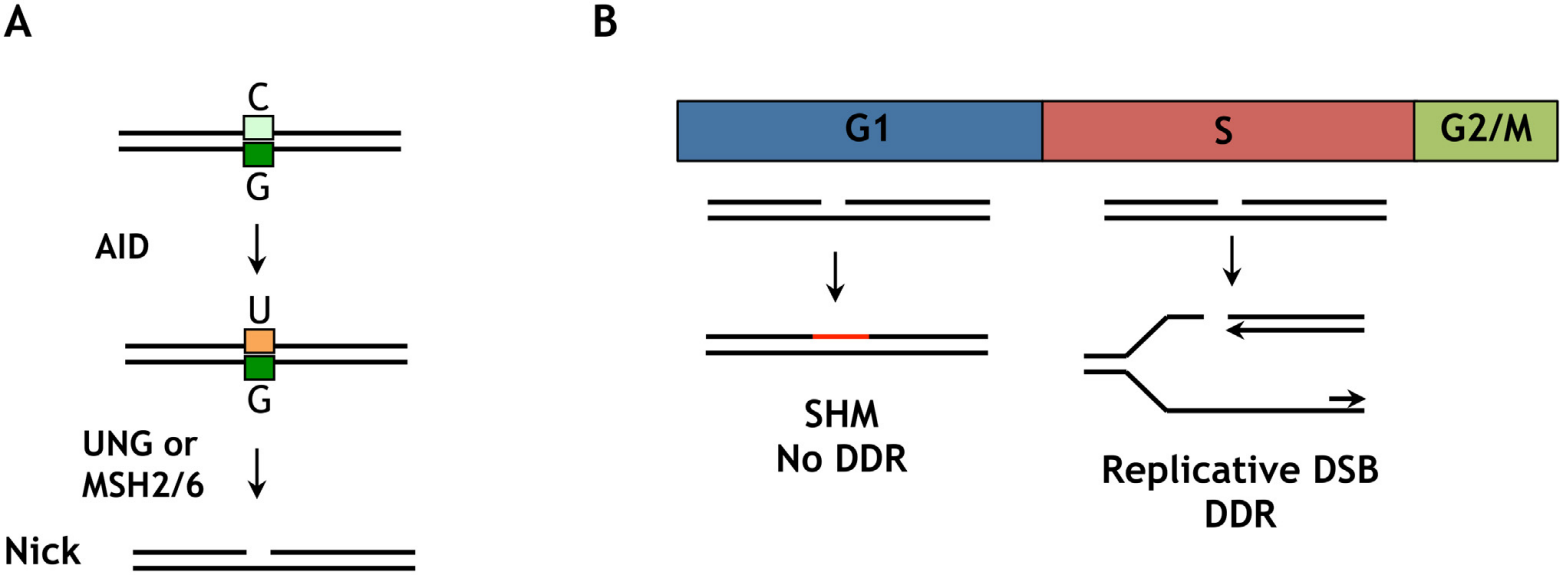
Distinct outcomes of an AID-initiated DNA nick in G1 and S/G2M phases. (A) Deamination of a C/G base pair by AID creates a U/G mispair at which UNG2 or MSH2/MSH6 will act to create a DNA nick. In either case, the repair processes that remove U generates a DNA nick as an intermediate. (B) In G1 phase, a nick can undergo mutagenic repair resulting in SHM. In S phase, if replication occurs before repair the nick will be converted to a replicative DSB. This may activate ATM and DNA-PK, cause γ-H2AX accumulation, a DNA damage response, checkpoint activation and potential loss of viability.

G1 phase nuclear AID accelerates SHM and CSR without compromising cell viability in G1 phase, but is not well-tolerated in S-G2/M phases. The ability of G1 phase cells to tolerate nuclear AID may reflect the relative efficiency of repair of AID-initiated nicks in G1 phase compared to later phases of cell cycle, as well as the potential for nicks to be converted to potentially deleterious replicative DSBs in S phase (Fig 7). AID deaminates C to generate U, which is repaired by UNG2- or MSH2/6-dependent pathways which generate nicks (single-strand breaks) as critical intermediates (Fig 7A). The evidence that Ig gene diversification occurs in G1 phase [28–31] suggests that unrepaired nicks do not persist into S phase. DNA replication in S phase will convert a nick into a replicative DSB (Fig 7B), which can initiate a DNA damage response and activate checkpoints that prevent cell cycle progression [48]. S phase nuclear AID is therefore a potential source of replicative DSBs, and if these exceed the capacity for repair they can compromise cell proliferation.

The resilience of G1 phase cells to AID-initiated damage was especially evident in the AID^F193A^-mCherry-CDT1 transductants. The design of this construct completely restricted nuclear AID to G1 phase, by combining the effect of the CDT1 tag with the F193A mutation, which prevents exported AID from re-entering the nucleus in S-G2/M phase. In contrast, other AID derivatives designed to accelerate SHM have exhibited a negative impact on cell viability, including other NES mutants [26,47], AID mutants selected for increased deamination activity [25,26,47] and the naturally occurring human AIDΔE5 dominant negative mutant [27].

Reduced viability was associated with the presence of nuclear AID in S phase, in AID^F193A^-mCherry and AID^F193A^-mCherry-GEM transductants (Fig 6D and 6E). We note that even though the GEM tag was predicted to restrict nuclear protein to S-G2/M phases, there was no nuclear AID-mCherry-GEM signal in any stage of cell cycle. In light of the evidence that nuclear AID is degraded more slowly in G1 than S or G2/M phase (Fig 2), it is likely that the AID-mCherry-GEM fusion protein was eliminated from the nucleus in G1 phase by degradation targeted to the GEM tag, and in S phase by degradation targeted to AID itself. AID has eight lysine targets for ubiquitination [20], and differential ubiquitination may be one source of temporal regulation.

Cell cycle regulation of the error-prone pathways that promote SHM may enhance repair of AID-initiated damage in G1 phase, and also explain some apparent differences in the SHM spectra of the different transductants. An especially high fraction of transversion mutations from G to T was evident in AID-mCherry-GEM transductants (11.1%) relative to AID-mCherry (0%) or AID-mCherry-CDT1 transductants (3.4%; Fig 5D), a class of mutations that can be generated by activity of Rev1 [49] or Polη [50]. In addition, a reduced frequency of mutations at A and T was evident in AID-mCherry-CDT1 (6.8%) and AID-mCherry-GEM (8.4%) relative to AID-mCherry transductants (17.9%; Fig 5). The reduced frequency of mutations at A and T argues against a predominant role for Polη, which is especially active at mutating at A and T [51], and makes Rev1 a more likely candidate for the G to T transversions in AID-mCherry-GEM transductants. This suggests that Rev1 may function late in cell cycle. Consistent with this, Rev1 has been shown to repair UV damage at gaps that persist into G2 phase [52].

The CDT1 tag destabilizes nuclear protein outside G1 phase [32] and would not be predicted to increase nuclear abundance at any stage of cell cycle. Nonetheless, AID-mCherry-CDT1 nuclear signal exceeded that of AID-mCherry (Fig 4C). This somewhat paradoxical result could be explained if the CDT1 tag prevented cytoplasmic retention [19] or enabled more efficient nuclear import. Consistent with the latter possibility, treatment with LMB or LMB+MG132 did cause a slightly more rapid increase in nuclear signal in AID-mCherry-CDT1 than AID-mCherry transductants (S7 Fig). In addition, AID may be regulated by feedback loops that determine nuclear levels in G1 phase based on the level in another compartment or stage of cell cycle. In that case, low levels of AID in G2/M phase may lead to elevated nuclear levels in the next G1 phase, as was evident in the AID-mCherry-CDT1 transductants (Fig 4D), favoring a cell that has not carried out Ig gene diversification in one cell cycle to do so in the next.

### Phosphorylation and catalytic activity regulate AID abundance

AID Ser3 is a site of negative regulation, and phosphorylation at this position was previously shown to downregulate SHM and CSR [34]. By analysis of the phosphomimetic mutant, AID^S3D^, we showed that modification at this position regulates AID nuclear abundance and the rate of AID nuclear degradation in G1 phase (Fig 3). Phosphorylation at this site is modulated by the protein phosphatase, PP2A, a key signaling molecule, targets of which include cell cycle regulators.

AID abundance also appears to be regulated by its catalytic activity. Both the nuclear and cytoplasmic signals of the AID^H56A^-mCherry catalytic mutant were well below signals of wild type AID-mCherry (Fig 3). The Ramos AID^H56A^-mCherry transductants express endogenous AID and hypermutate actively, so the reduced signal of the catalytic mutant must reflect a protein-intrinsic mechanism directed to the mutant protein. It is possible that sort of quality control mechanism may contribute to AID stability. Catalytically inactive AID may, for example, be a better target for ubiquitination.

The possibility that AID might be regulated by a feedback mechanism that reported on AID-induced DSBs was previously raised by Chaudhuri and collaborators, who claimed that a catalytically inactive AID double mutant, AID^H56R E58Q^, was a poor substrate for Ser38 phosphorylation in B cells carrying out CSR [53]. Central to that claim was evidence of apparently reduced recovery of the Ser38-phosphorylated derivative of AID^H56R E58Q^. AID^H56R E58Q^ bears amino acid substitutions at two residues at the catalytic active site. Reduced abundance of the AID^H56R E58Q^ was evident in data included in supplementary data accompanying that report but not commented on by the authors [53]. Our evidence for reduced abundance of the AID^H56A^-mCherry catalytic mutant (Fig 3) provides an alternative explanation for poor recovery of Ser38-phosphorylated AID^H56R E58Q^. It would be appropriate to revisit the claim that an ATM-dependent signal necessary for AID Ser38 phosphorylation requires DNA damage for induction [53] in experiments in which results are subjected to more rigorous quantification.

Several groups have reported that treatments that cause DSBs (ionizing radiation, etoposide) can induce nuclear localization of AID [24,53,54]. A DSB-dependent signal may be relevant to CSR, but unlikely to be relevant to SHM. The DNA intermediates in CSR carry DSBs, which initiate a DNA damage response mediated by γ-H2AX, which is essential to CSR [41,55]. In contrast, SHM intermediates contain DNA nicks, not DSBs (Fig 7); and γ-H2AX is not essential for SHM [56] or induced in hypermutating B cells.

### Cell cycle tags are a powerful and practical tool

The CDT1 and GEM tags can be readily adapted to study repair in other contexts, and they also have potential practical utility. It has long been apparent that AID-initiated mutagenesis coupled with selection can in principle be used to optimize affinity or specificity of a variety of target proteins, including B and T cell receptors. However the complex regulation of AID has previously thwarted attempts to do so effectively. AID^F193A^-mCherry-CDT1 and analogous AID derivatives should be useful in strategies that seek to evolve antibodies and other proteins ex vivo by iterative cycles of hypermutation and selection. Cell cycle tags may also be useful for optimizing activities of enzymes that act on DNA or RNA in the nuclease, including CRISPR/Cas9 and other nucleases used for genome engineering and gene correction.

## Materials and Methods

### Ethics statement

All experiments involving primary murine B cells were approved by the University of Washington Institutional Animal Care and Use Committee.

### Expression constructs

The pEGFP-N3 construct for expression of AID-GFP was a gift from Dr. Javier Di Noia (Department of Microbiology and Immunology, University of Montreal, Montreal, Quebec, Canada). We substituted mCherry for a region of GFP flanked by ApaI and BsrGI restriction sites in the pEGFP-N3 construct to generate an AID-mCherry expression construct, pAID-mCh. Cell cycle reporter constructs p-mKO2-CDT1 CSII and p-mAG-GEM CSII, in a lentiviral vector, were a gift from Dr. Atsushi Miyawaki (Brain Science Institute, RIKEN, Hirosawa, Wako-city, Saitama 351–0198, Japan)

pAID-mCh CSII: We amplified AID-mCherry from pAID-mCh with primers PQL31, 5’-ATATCAATTGAGATCCCAAATGGACAGCC-3’ and PQL32, 5’-ATATTCTAGATTACTTGTACAGCTCGTCCATGC-3’, and inserted it between EcoRI and XbaI sites in p-mAG-GEM CSII, thereby replacing mAG-GEM with AID-mCherry.

pAID-mCh-CDT1 and pAID-mCh-GEM: We amplified CDT1 with primers PQL44 5’-TATATGTACAAGGGATATCCATCACACTGGCGGCC-3’ and PQL45 5’-TATATGTACATCTAGATTAGATGGTGTCCTGGTCC-3’ from p-mKO2-CDT1 CSII, and GEM with primers PQL44 5’-TATATGTACAAGGGATATCCATCACACTGGCGGCC-3’ and PQL46 5’- TATATGTACATCTAGATTACAGCGCCTTTCTCCG-3’ from p-mAG-GEM CSII, and inserted the resulting fragments between BsrGI and XbaI restriction sites of pAID-mCh CSII.

pAID-mKO2-CDT1 and pAID-mKO2-GEM: We amplified mKO2 with primers mKO2 FOR 5’-ATATGGATCCATCGCCACCATGGTGAGTGTG-3’ and mKO2 REV 5’-ATATGCGGCCGCCAGTGTGATGGATATCCGC-3’, and inserted the resulting fragment between BamHI and NotI restriction sites in pAID-mCh-CDT1 or pAID-mCh-GEM CSII, respectively.

pAID^F193A^-mCh-CDT1, pAID^F193A^-mCh-CDT1 and pAID^F193A^-mCh-GEM: F193A mutants were generated using *QuikChange II XL Site*-*Directed Mutagenesis Kit (Agilent) with primer set*, F193A FOR 5’-CTTACGAGACGCAGCTCGTACTTTGGGAC-3’ and F193A REV 5’-GTCCCAAAGTACGAGCTGCGTCTCGTAAG-3’.

pAID^S3A^-mCh, pAID^S3D^-mCh, pAID^S38A^-mCh, pAID^S38D^-mCh, pAID^H56A^-mCh, pAID^H56R^-mCh: Mutants were generated using *QuikChange II XL Site*-*Directed Mutagenesis Kit (Agilent)* with primer sets, S3A FOR 5’- GATCCCAAATGGACGCCCTCTTGATGAACC -3’ and S3A REV 5’- GGTTCATCAAGAGGGCGTCCATTTGGGATC -3’; S3D FOR 5’- GATCCCAAATGGACGACCTCTTGATGAAC -3’ and S3D REV 5’- GTTCATCAAGAGGTCGTCCATTTGGGATC -3’; S38A FOR 5’- GTGAAGAGGCGTGACGCTGCTACATCCTTTTC -3’ and S38A REV 5’- GAAAAGGATGTAGCAGCGTCACGCCTCTTCAC -3’; S38D FOR 5’- GTGAAGAGGCGTGACGATGCTACATCCTTTTC -3’ and S38D REV 5’- GAAAAGGATGTAGCATCGTCACGCCTCTTCAC -3’; H56A FOR 5’- GAACGGCTGCGCCGTGGAATTGCTC -3’ and H56A REV 5’- GAGCAATTCCACGGCGCAGCCGTTC -3’; H56R FOR 5’- GAACGGCTGCCGCGTGGAATTGCTC -3’ and H56R REV 5’- GAGCAATTCCACGCGGCAGCCGTTC -3’. The H56A catalytic mutant was shown to lack ability to initiate SHM using the sIgM loss assay (S8 Fig).

### Lentivirus production

Lentiviral particles were produced using second-generation packaging plasmids in 293T cells. 293T cells were transfected with transfer vector, viral packaging vector (psPAX2), and viral envelope vector (pMD2G) at 4:2:1 ratio using Lipofectamine LTX (Life Technologies, Cat # 15338100) transfection as directed by manufacturer’s protocol. Viral particles were collected at 24hr and 48hr after transfection and passed through 0.22 μm membrane (Steriflip; EMD Millipore; Cat # SCGP00525). Virus particles were used without further concentration.

### Cell culture and transduction

The human Burkitt lymphoma cell line, Ramos, was cultured in supplemented RPMI 1640 (Gibco), which contained 10% FBS, 2 mM L-glutamine, penicillin/ streptomycin, 1X non-essential amino acids (Gibco), 1 mM sodium pyruvate, and 10 mM HEPES. Lentiviral transductions used 2x10^5^ cells cultured in medium containing 8 μg/ml of polybrene. Following transduction, cells were cultured for 3–4 days and these recent transductants then sorted for mCherry+ to enrich for transduced cells, typically constituting 0.1–10% of the population. Cells were treated with leptomycin B (LMB; LC Laboratories) at 50 ng/ml and MG132 (Z-Leu-Leu-Leu-aldehyde; Sigma-Aldrich) at 50 μM.

### Assays of cell cycle and viability

To determine cell cycle distribution by flow cytometry, cells were fixed, permeabilized with 0.5% Triton X-100, stained with DAPI (2 μg/ml) and analyzed by FACS. Viable cells were counted after trypan blue staining. Cell viability was confirmed by CellTiter-Glo Luminescent Cell Viability Assay (Promega).

### High content screening (HCS) microscopy and analysis

Cells were fixed in 3.7% formaldehyde at a density of 2x10^6^ cells/ml and stained with whole cell stain (HCS CellMask, Invitrogen) and DAPI (0.2 μg/ml). Fixed cells were then washed, resuspended in PBS and spun down in a 96-well μclear microplate (Greiner Bio One) for imaging. Cells were imaged by Thermo Scientific ArrayScan VTI HCS reader, analyzing 3000–6000 cells in each treatment group. Cells with very low or very high mCherry signals were eliminated, gating based on the mock transduction control (low) and eliminating cells with signals more than 5 SD from the mean (high). Signal as determined by HCS is presented in arbitrary units, facilitating direct comparisons among different samples and different datasets.

The HCS Colocalization BioApplication protocol was used to determine nuclear and whole cell boundaries in individual cells as defined by DAPI and HCS CellMask, respectively, thereby defining the cytoplasmic region as the region between nuclear and whole cell boundaries. The average signal in the nuclear and cytoplasmic compartments was determined in individual cells by measuring the total intensity of mCherry signal divided by area within each compartment. The ratio of nuclear to cytoplasmic signal (N/C) was calculated as the ratio of the average signals of nuclear and cytoplasmic mCherry.

Confocal microscopy showed that AID-mCherry was mostly absent from the nucleus when out-of-focus signal was eliminated, regardless of the level of cytoplasmic signal (S13 Fig). HCS analysis of AID-mCherry transductants showed that nuclear AID-mCherry signals increased linearly with increasing cytoplasmic signals (slope of linear regression = 0.848; S14 Fig), consistent with a contribution of cytoplasmic signal from above or below the nucleus to the signal identified as nuclear by HCS. Thus in order to enable accurate comparisons of nuclear signals among AID-mCherry, AID-mCherry-CDT1, AID-mCherry-GEM, AID^F193A^-mCherry, AID^F193A^-mCherry-CDT1, and AID^F193A^-mCherry-GEM transductants, the nuclear signal for each cell, as determined by HCS, was corrected by subtraction of the corresponding baseline value, as established by linear regression analysis of nuclear vs. cytoplasmic signals of untreated AID-mCherry transductants (S11 Fig), using the formula: Nuclear signal = (Nuclear signal)_HCS_ - (0.848X +21.1).

### Assays of sIgM loss frequency in Ramos B cell transductants

sIgM loss frequency provides a convenient surrogate assay for SHM (1, 2). To determine fractions of sIgM- cells, 2-5x10^5^ cells were fixed in 3.7% formaldehyde and stained with anti-human IgM (1:500, Southern Biotech), and sIgM- variants quantified by FACS as described [46]. To establish that selective pressure was not sufficient to affect the frequency of sIgM loss, we assayed loss of mCherry signal posttransduction (S15 Fig). There was modest loss of mCherry expression between days 3 and 7 in the AID-mCherry-CDT1 transductants (decrease from 37.2% to 31.3%), consistent with some selective pressure against AID-mCherry-CDT1 expression, but not sufficient to alter interpretation of the sIgM loss data.

### Assay of CSR in primary splenic B cells

B cells were isolated from spleens of C57BL/6 mice and enriched through a negative selection in AUTOMACs with biotinylated anti-CD43 antibody (BD Pharmigen, Cat # 5532269) and streptavidin magnetic microbeads (Miltenyi Biotech, Cat # 130-048-102). Purified B cells were transduced for 24 hr in X-vivo medium (Lonza) containing 2 mM L-glutamine, 50 μM ß-mercaptoethanol, 5 ng/ml IL-4 (R&D Systems, cat# 404-ML-010) and 1 μg/mL anti-CD40 antibody (BioLegend, Cat# 102802) in 100 μl total volume in a round bottom 96-well plate, then transferred at 24 hr to supplemented RPMI (see above) containing 5 ng/ml IL-4 and 1 μg/ml anti-CD40 antibody. Cells were cultured for 4–5 days, stained with anti-IgG1 (FITC anti-mouse IgG1; BioLegend, Cat# 406605), and surface IgG1 quantified by flow-cytometry.

### Single-cell PCR and sequencing of V_H_ regions

At day 7 post sorting recent transductants for mCherry+ cells, single cells from AID-mCherry, AID-mCherry-CDT1 or AID-mCherry-GEM transductant populations were aliquoted, one cell per well, into 96-well plates containing 20 μl of Pfu reaction buffer (Agilent). Samples were frozen, thawed, and treated with 250 μg/ml proteinase K for 1 hr at 50°C then 5 min at 95°C, the primers and high-fidelity Pfu Turbo DNA polymerase (Agilent) were added and the rearranged V_H_ region amplified by nested PCR with first round primers, RV_H_FOR QL 5’-TCCCAGGTGCAGCTACAGCAG-3’ and JOL48 QL 5’-GTACCTGAGGAGACGGTGACC-3’ [57]; followed by 1:30 dilution and second round amplification with primers 5’-AGGTGCAGCTACAGCAGTG-3’ and 5’-GCCCCAGACGTCCATACC-3’. Predicted sizes of PCR products were confirmed by gel electrophoresis and fragments purified and sequenced.

## Supporting Information

S1 Fig Cell cycle profile of Ramos B cells is unaltered by treatment with MG132, LMB, or MG132+LMB treatment in Ramos B cells. Representative cell cycle profiles of Ramos B cell AID-mCherry transductants following treatment with MG132, LMB, or MG132+LMB for indicated time. Estimated percentage of cells in G1, S, and G2/M phase (as determined by the Watson Pragmatic computational model in FlowJo) is tabulated below each cell cycle profile.
(TIFF)

S2 Fig HCS assessment of DNA content; nuclear, cytoplasmic and whole cell area and total and average signals in G1, S and G2/M phase Ramos B cell AID-mCherry transductants. (A) Representative cell cycle profile for untreated Ramos B cell AID-mCherry transductant populations, showing fractions identified as G1, S, and G2/M populations. Cell cycle phase was determined based on DNA content as measured by total intensity of DAPI staining. Cells were ranked based on DNA content, and ranks 1–4 assigned to G1 phase, ranks 10–16 to S phase, and ranks 21–24 to G2/M phase. (B) Total intensity of mCherry signal per cell across DNA content. Error bars denote SEM of the population. (C) Average nuclear, cytoplasmic, and whole cell area for G1, S and G2/M phase Ramos B cell AID-mCherry transductant populations. Error bars denote SEM of the population and in some cases are too small to discern clearly. (D) Population average of total intensity of mCherry signal in the nuclear and cytoplasmic compartments and whole cells are shown for G1, S and G2/M phase in Ramos B cell AID-mCherry transductants. Error bars denote SEM of the population and in some cases are too small to discern clearly. (E) Population average of the average intensity of AID-mCherry expression in Ramos B cells in the nuclear and cytoplasmic compartments and whole cells are shown for G1, S and G2/M phase cells. Error bars denote SEM of the population and in some cases are too small to discern clearly.
(TIFF)

S3 Fig Kinetics of response of AID-mCherry to treatment with LMB. Three independent experiments analyzing kinetics of response of AID-mCherry nuclear (solid lines) and cytoplasmic (dashed lines) signals to treatment with LMB in G1, S and G2/M phase cells. Dotted line represents no change (fold change of 1). Each point represents a population average, and black bars represent SEM of the population, which are too small to discern. These data and those shown in Fig 2A were used to calculate cell cycle-dependent differences in nuclear stability of AID-mCherry (Fig 2B).
(TIFF)

S4 Fig Decreased abundance of AID catalytic mutants. Flow cytometry of Ramos AID-mCherry, AID^56A^-mCherry, AID^56R^-mCherry and mock transductants, showing cell number relative to mCherry signal. Flow cytometry of Ramos AID-mCherry, AID^56A^-mCherry, AID^56A-H^-mCherry (revertants) and mock transductants, showing cell number relative to mCherry signal.
(TIFF)

S5 Fig Cell cycle dependence of abundance of AID mutants. Nuclear, cytoplasmic and whole cell mCherry signal of AID bearing mutations at indicated residues, in G1, S, or G2/M phase cells. Signal was determined by HCS (see Methods).
(TIFF)

S6 Fig CDT1 and GEM tags confer cell cycle-dependent restriction of nuclear stability to fluorescent reporter proteins. (A) Representative fluorescence images of Ramos mKO2-CDT1 and Ramos mAG-GEM transductants, showing mKO2 or mAG, DAPI and merged signals. (B) Flow cytometry of Ramos mKO2-CDT1 and mAG-GEM transductants, showing cell number relative to DNA content and percent of cells in G1 or S-G2/M phases (above), and mKO2 or mAG signal and fraction of population in each quadrant (below).
(TIFF)

S7 Fig Destabilization and redistribution of AID-mCherry, AID-mCherry-CDT1, and AID-mCherry-GEM upon treatment with MG132, LMB, or both. Quantification of nuclear and cytoplasmic AID-mCherry signal and N/C ratio in treated relative to untreated cell populations at indicated times post-treatment with MG132, LMB, or both in Ramos B cells expressing AID-mCherry, AID-mCherry-CDT1, or AIDmCherry-GEM. Each point on the graph represents the population average, and black bars are SEM of the population.
(TIFF)

S8 Fig sIgM loss assays. (A) Representative FACS profiles of Ramos AID-mCherry, AID-mCherry-CDT1, AID-mCherry-GEM, AID^H56A^-mCherry and mock transductants at day 7 after sorting mCherry+ cells among recent transductants. Above, mCherry signal gated relative to mock transductants, indicating percentage of mCherry+ cells. Below, sIgM staining profiles, from gate shown above, of mCherry+ cells for AID-mCherry, AID-mCherry-CDT1, and AID-mCherry-GEM transductants; and of mCherry- cells for mock transductants. Percentage of sIgM- cells is shown. (B) Above, flow cytometry of indicated AID-mKO2-CDT1 or AID-mKO2-GEM transductants, showing cell number relative to DNA content and percent of cells in G1 or S-G2/M phases (above), and mKO2 signal and fraction of population in each quadrant (below). Below, representative FACS profiles of AID-mKO2-CDT1, AID-mKO2-GEM and mock transductants at day 7 after sorting recent transductants for mKO2+ cells. Above, mKO2 signal gated relative to mock transductants, indicating percentage of mKO2+ cells. Below, sIgM staining profiles, from gate shown above, of mKO2+ cells for AID-mKO2-CDT1 and AID-mKO2-GEM transductants; and of mKO2- cells for mock transductants. Percentage of sIgM- cells is shown.
(TIFF)

S9 Fig AID-mCherry CDT1 accelerates CSR in primary murine B cells. (A) Expression level of AID-mCherry transductants showing MFIs of mock transductants and mCherry+ cells among AID-mCherry transductants. (B) Flow cytometry of indicated transductants of primary murine splenic B cells, showing percent of cells that are mCherry+ (above) and fraction of IgG1+ cells among mCherry+ cells (below) at day 4 post transduction. (C) Flow cytometry of indicated transductants of primary murine splenic B cells, showing percent of cells that are mCherry+ (above) and fraction of IgG1+ cells among mCherry+ cells (below) at day 5 post transduction.
(TIFF)

S10 Fig Sequence analysis of rearranged IgV_H_ regions in single cells. The parental nucleic acid sequence is shown in black, with positions of nucleotides numbered starting from the first base of first codon, corresponding amino acids are shown below each codon, and CDR1 and CDR2 underlined. Above the parental sequence, point mutations are shown in red, deletions as black bars and insertions as open triangles. Only sequences with unique mutation spectrum are shown.
(TIFF)

S11 Fig Mutations in V_H_ regions. Percent of point mutations, deletions, and insertions in mutated V_H_ regions of AID-mCherry, AID-mCherry-CDT1, or AID-mCherry-GEM transductants.
(TIFF)

S12 Fig Quantification of cell viability of AID^F193A^-mCherry, AID^F193A^-mCherry-CDT1 and AID^F193A^-mCherry-GEM transductants. (A) Cell viability of indicated transductant populations, as determined by trypan blue exclusion. These independent populations were cultured at lower (Expt. a) and higher (Expt. b) density than the experiment shown in the text (Fig 5E), to ensure that cell density did not account for differences in relative viability. Viability was determined at the indicated day after sorting mCherry+ cells among recent transductants. (B) Cell viability of indicated transductant populations, as determined by assaying ATP levels at days 7 and 11 post-sorting mCherry+ cells among recent Ramos transductants. Viability of the population shown was also analyzed by trypan blue exclusion, and those in Expt. b in panel A, above.
(TIFF)

S13 Fig Analysis of nuclear AID-mCherry signals by confocal microscopy. (A) Fluorescence images of AID-mCherry transductants acquired by confocal fluorescent microscopy. DAPI (left), mCherry (middle) and merge (right) signals are shown. (B) Representative individual AID-mCherry transductants (1–4 in image on left) and plot files of their mCherry fluorescence intensities along arbitary lines as indicated. Note the range of maximum fluorescence intensities.
(TIFF)

S14 Fig Correction of HCS nuclear signal correction for the contribution of cytoplasmic signal. Scatter plot of nuclear vs. cytoplasmic mCherry signals of Ramos AID-mCherry transductants. Dashed line represents the linear model obtained from linear regression analysis. Right, the equation for the linear model is shown. Nuclear signals as determined by HCS were corrected for cytoplasmic baseline using the formula shown (see Materials and Methods).
(TIFF)

S15 Fig Cell cycle and expression profiles of Ramos transductants at days 3 and 7 post sort. Flow cytometry of Ramos AID-mCherry, AID-mCherry-CDT1, AID-mCherry-GEM, AID^F193A^-mCherry, AID^F193A^-mCherry-CDT1 and AID^F193A^-mCherry-GEM transductants, showing cell number relative to DNA content and percent of cells in G1 or S-G2/M phases (left), and mCherry signal and fraction of population in each quadrant (right) for day 3 and day 7 post sort.
(TIFF)

S1 Table Probability tests for Fig 1D. Statistical tests were performed using two-tailed, unpaired Student’s t-test, assuming unequal variances, for comparison of nuclear and cytoplasmic AID-mCherry signal and the N/C ratio between different treatment groups and between different times post treatment and untreated control in each treatment group.
(DOCX)

S2 Table Probability test for Fig 2A: Cell Cycle Comparisons. Statistical tests were performed using two-tailed, unpaired Student’s t-test, assuming unequal variances, for comparison of nuclear and cytoplasmic AID-mCherry signal and the N/C ratio between G1 and S; G1 and G2/M; and S and G2/M at different times post-treatment in each treatment group.
(DOCX)

S3 Table Subcellular distribution of AID determined by HCS microscopy. The number of cells (N) and the mean total, cytoplasmic, and nuclear mCherry signals are tabulated for Ramos AID-mCherry, AID-mCherry-CDT1, AID-mCherry-GEM, AID^F193A^-mCherry, AID^F193A^-mCherry-CDT1 and AID^F193A^-mCherry-GEM transductants. Nuclear signals as determined by HCS were corrected for cytoplasmic baseline (see Materials and Methods). Statistical tests were performed using two-tailed, unpaired Student’s t-test, assuming unequal variances for comparisons among transductant populations.
(DOCX)

S4 Table Cell cycle dependence of subcellular localization of AID. The number of cells (N) and the mean total, cytoplasmic, and nuclear mCherry signals are tabulated for G1, S and G2/M cells in Ramos AID-mCherry, AID-mCherry-CDT1 and AID-mCherry-GEM, AID^F193A^-mCherry, AID^F193A^-mCherry-CDT1, AID^F193A^-mCherry-GEM transductant populations. Nuclear signals as determined by HCS were corrected for cytoplasmic baseline (see Materials and Methods). Statistical tests were performed using two-tailed, unpaired Student’s t-test, assuming unequal variances for comparisons among G1, S and G2/M phase cells in transductant populations.
(DOCX)
