# Supplementary figures and images for: Cell Cycle Regulates Nuclear Stability of AID and Determines the Cellular Response to AID
Source: PLoS Genet. 2015 Sep 10;11(9):e1005411. doi: 10.1371/journal.pgen.1005411 (PMC4565580; doi:10.1371/journal.pgen.1005411)

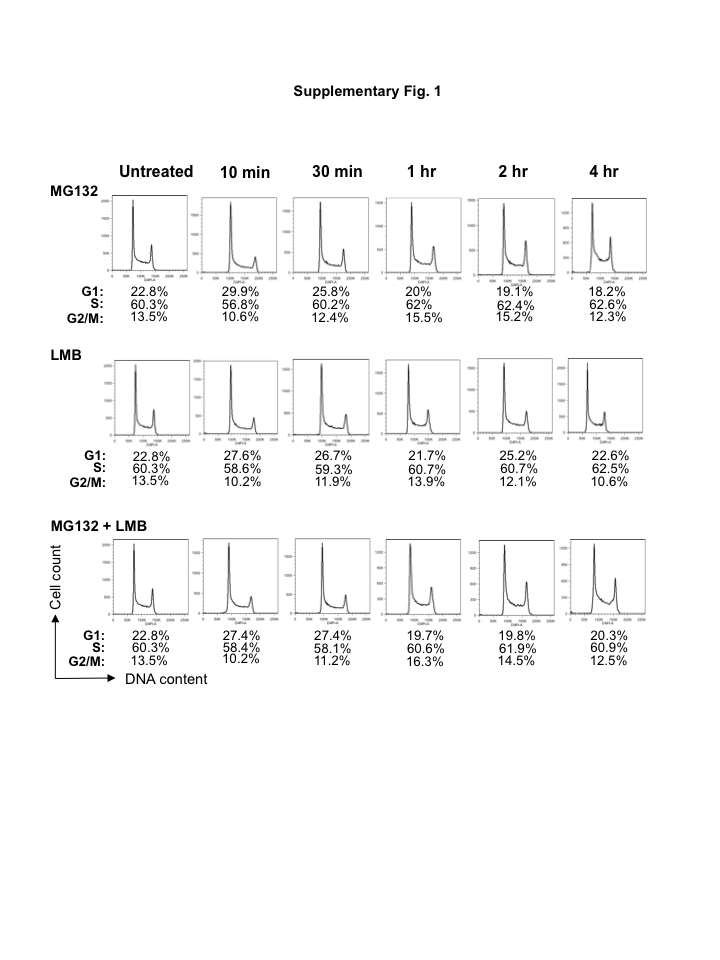

Supplement: S1 Fig — Representative cell cycle profiles of Ramos B cell AID-mCherry transductants following treatment with MG132, LMB, or MG132+LMB for indicated time. Estimated percentage of cells in G1, S, and G2/M phase (as determined by the Watson Pragmatic computational model in FlowJo) is tabulated below each cell cycle profile. (TIFF) [file pgen.1005411.s001.tiff]

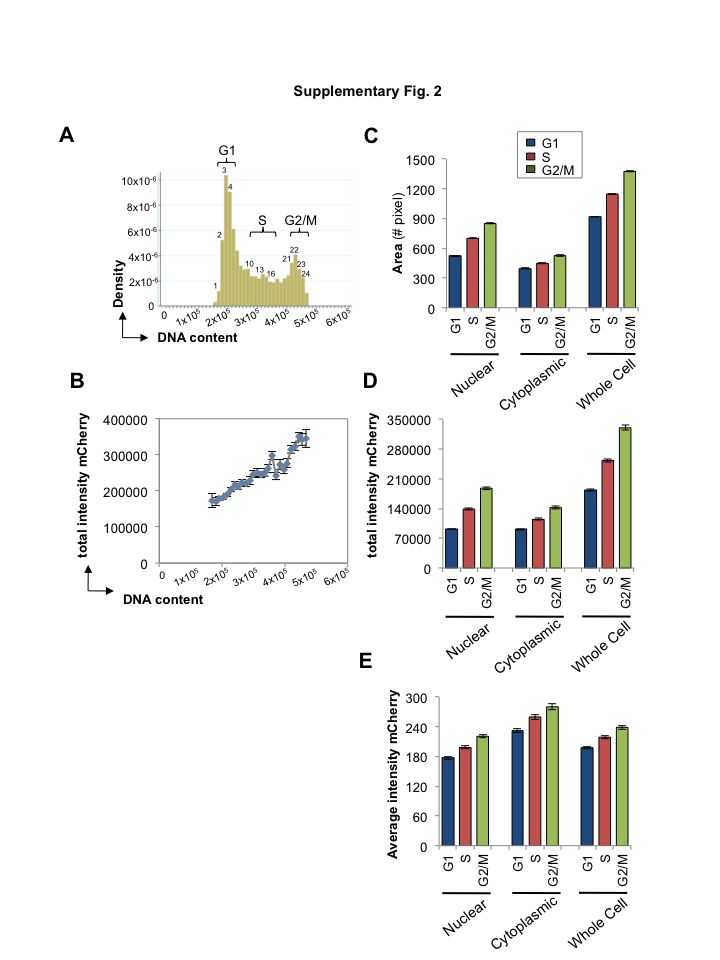

Supplement: S2 Fig — (A) Representative cell cycle profile for untreated Ramos B cell AID-mCherry transductant populations, showing fractions identified as G1, S, and G2/M populations. Cell cycle phase was determined based on DNA content as measured by total intensity of DAPI staining. Cells were ranked based on DNA content, and ranks 1–4 assigned to G1 phase, ranks 10–16 to S phase, and ranks 21–24 to G2/M phase. (B) Total intensity of mCherry signal per cell across DNA content. Error bars denote SEM of the population. (C) Average nuclear, cytoplasmic, and whole cell area for G1, S and G2/M phase Ramos B cell AID-mCherry transductant populations. Error bars denote SEM of the population and in some cases are too small to discern clearly. (D) Population average of total intensity of mCherry signal in the nuclear and cytoplasmic compartments and whole cells are shown for G1, S and G2/M phase in Ramos B cell AID-mCherry transductants. Error bars denote SEM of the population and in some cases are too small to discern clearly. (E) Population average of the average intensity of AID-mCherry expression in Ramos B cells in the nuclear and cytoplasmic compartments and whole cells are shown for G1, S and G2/M phase cells. Error bars denote SEM of the population and in some cases are too small to discern clearly. (TIFF) [file pgen.1005411.s002.tiff]

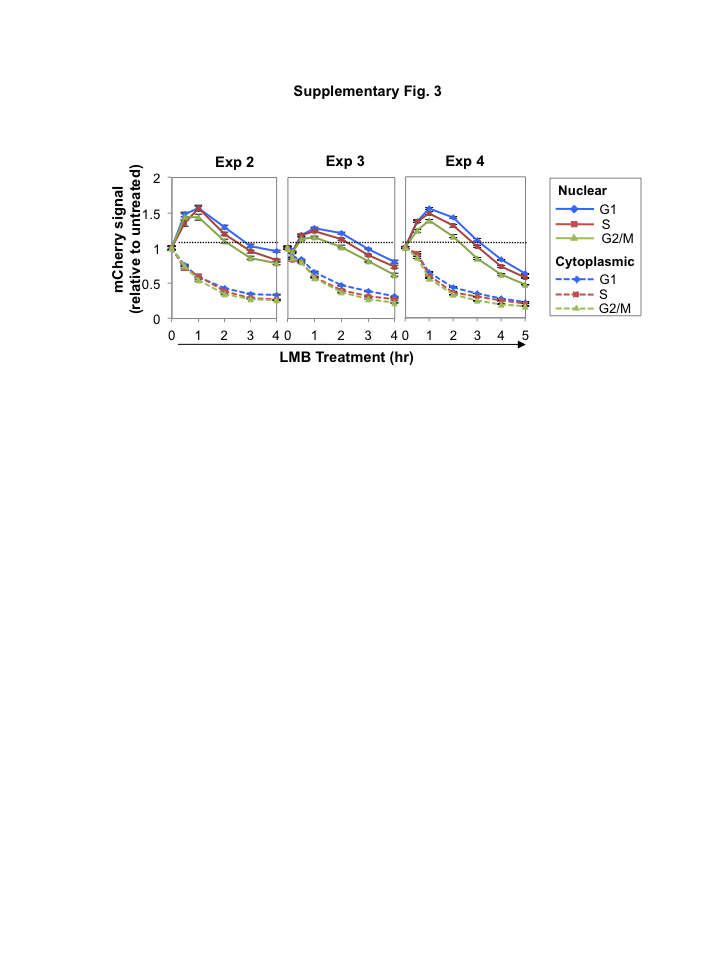

Supplement: S3 Fig — Three independent experiments analyzing kinetics of response of AID-mCherry nuclear (solid lines) and cytoplasmic (dashed lines) signals to treatment with LMB in G1, S and G2/M phase cells. Dotted line represents no change (fold change of 1). Each point represents a population average, and black bars represent SEM of the population, which are too small to discern. These data and those shown in Fig 2A were used to calculate cell cycle-dependent differences in nuclear stability of AID-mCherry (Fig 2B). (TIFF) [file pgen.1005411.s003.tiff]

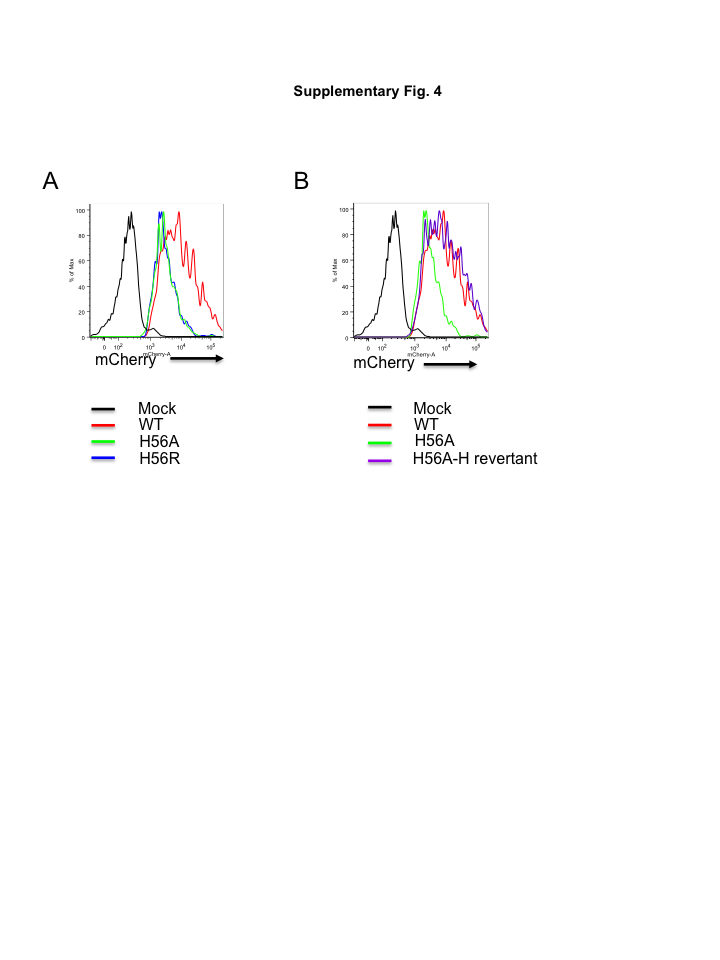

Supplement: S4 Fig — Flow cytometry of Ramos AID-mCherry, AID56A-mCherry, AID56R-mCherry and mock transductants, showing cell number relative to mCherry signal. Flow cytometry of Ramos AID-mCherry, AID56A-mCherry, AID56A-H-mCherry (revertants) and mock transductants, showing cell number relative to mCherry signal. (TIFF) [file pgen.1005411.s004.tiff]

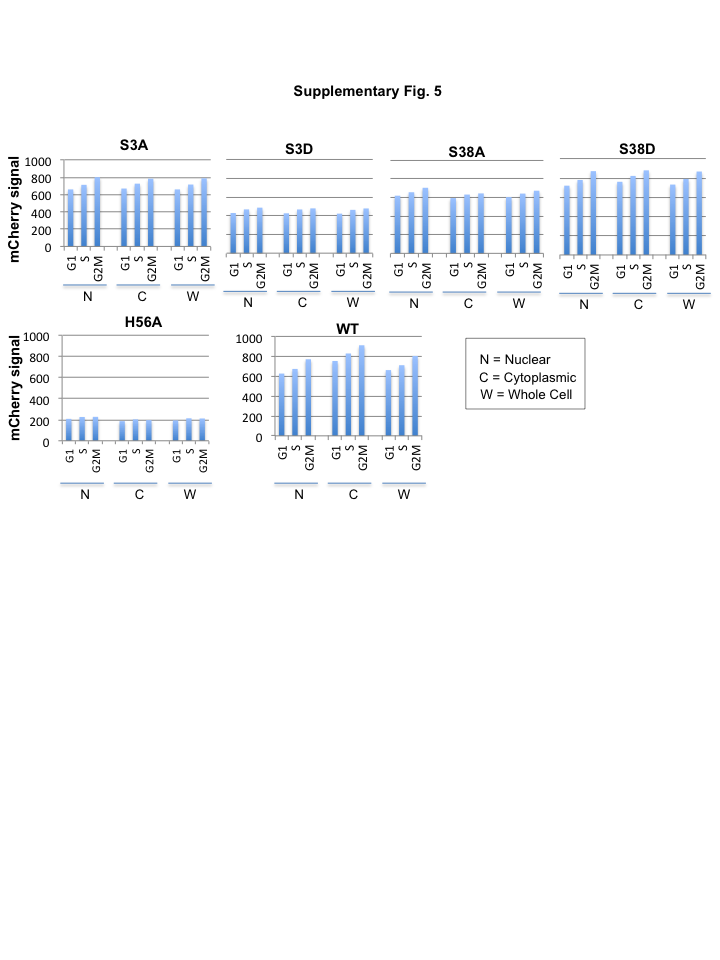

Supplement: S5 Fig — Nuclear, cytoplasmic and whole cell mCherry signal of AID bearing mutations at indicated residues, in G1, S, or G2/M phase cells. Signal was determined by HCS (see Methods). (TIFF) [file pgen.1005411.s005.tiff]

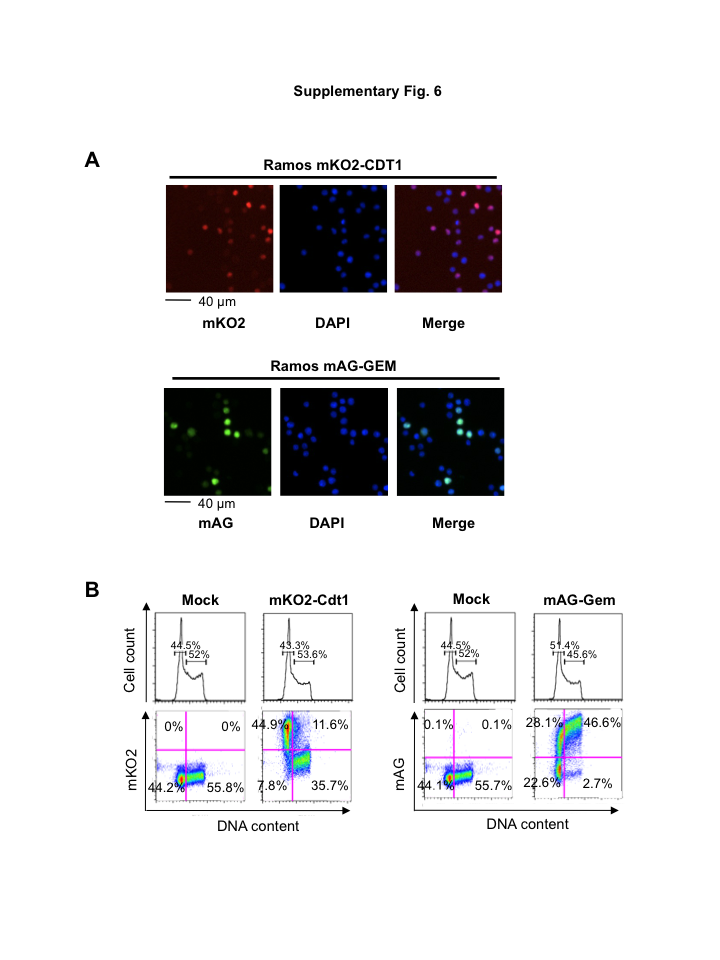

Supplement: S6 Fig — (A) Representative fluorescence images of Ramos mKO2-CDT1 and Ramos mAG-GEM transductants, showing mKO2 or mAG, DAPI and merged signals. (B) Flow cytometry of Ramos mKO2-CDT1 and mAG-GEM transductants, showing cell number relative to DNA content and percent of cells in G1 or S-G2/M phases (above), and mKO2 or mAG signal and fraction of population in each quadrant (below). (TIFF) [file pgen.1005411.s006.tiff]

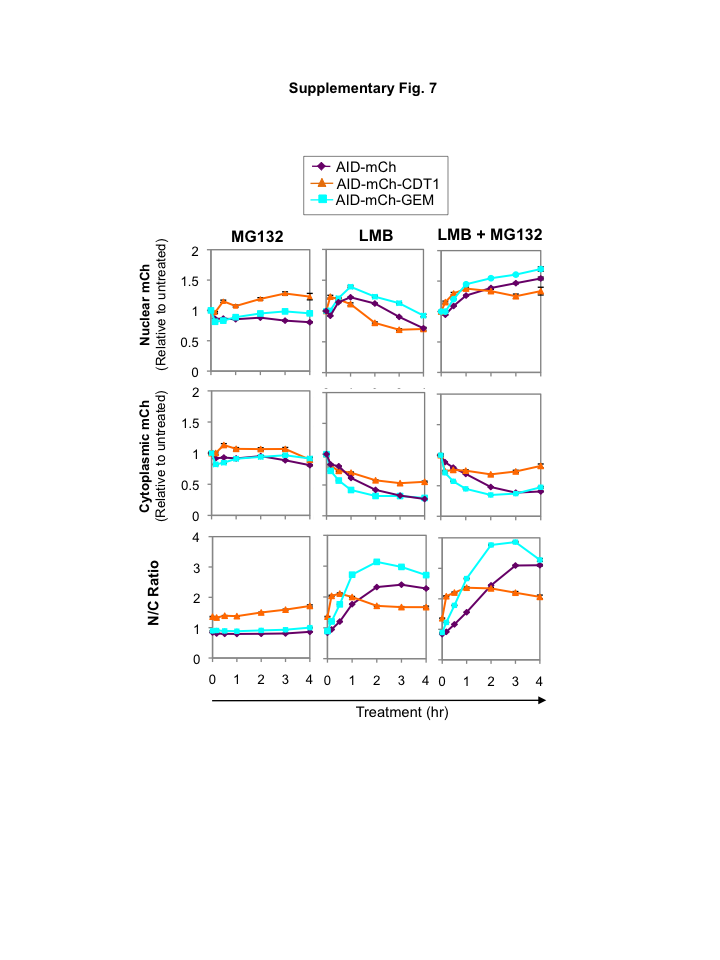

Supplement: S7 Fig — Quantification of nuclear and cytoplasmic AID-mCherry signal and N/C ratio in treated relative to untreated cell populations at indicated times post-treatment with MG132, LMB, or both in Ramos B cells expressing AID-mCherry, AID-mCherry-CDT1, or AIDmCherry-GEM. Each point on the graph represents the population average, and black bars are SEM of the population. (TIFF) [file pgen.1005411.s007.tiff]

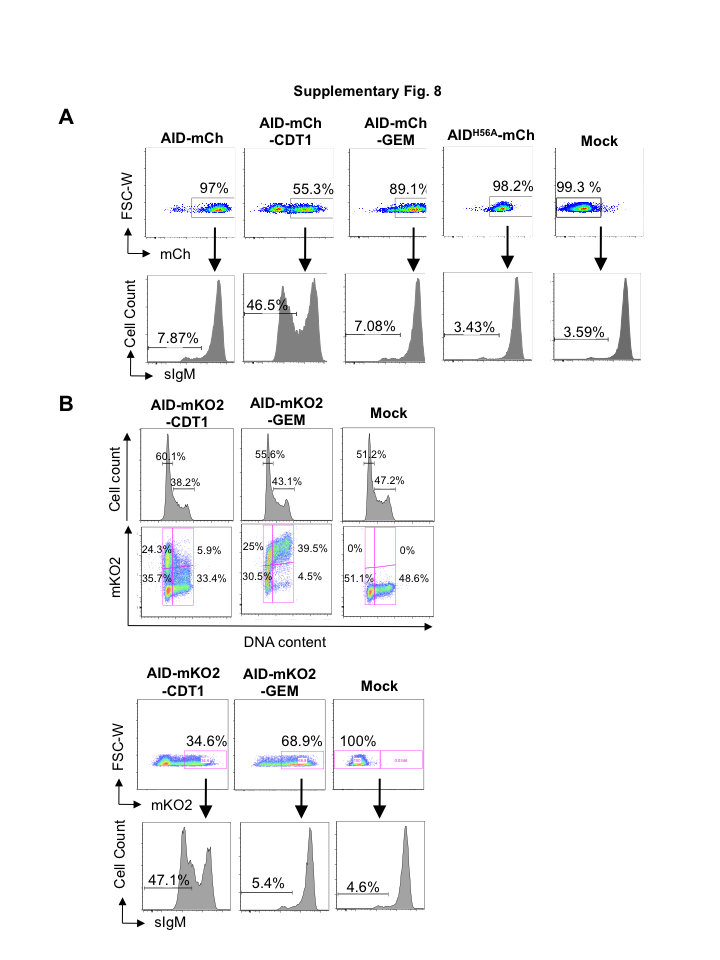

Supplement: S8 Fig — (A) Representative FACS profiles of Ramos AID-mCherry, AID-mCherry-CDT1, AID-mCherry-GEM, AIDH56A-mCherry and mock transductants at day 7 after sorting mCherry+ cells among recent transductants. Above, mCherry signal gated relative to mock transductants, indicating percentage of mCherry+ cells. Below, sIgM staining profiles, from gate shown above, of mCherry+ cells for AID-mCherry, AID-mCherry-CDT1, and AID-mCherry-GEM transductants; and of mCherry- cells for mock transductants. Percentage of sIgM- cells is shown. (B) Above, flow cytometry of indicated AID-mKO2-CDT1 or AID-mKO2-GEM transductants, showing cell number relative to DNA content and percent of cells in G1 or S-G2/M phases (above), and mKO2 signal and fraction of population in each quadrant (below). Below, representative FACS profiles of AID-mKO2-CDT1, AID-mKO2-GEM and mock transductants at day 7 after sorting recent transductants for mKO2+ cells. Above, mKO2 signal gated relative to mock transductants, indicating percentage of mKO2+ cells. Below, sIgM staining profiles, from gate shown above, of mKO2+ cells for AID-mKO2-CDT1 and AID-mKO2-GEM transductants; and of mKO2- cells for mock transductants. Percentage of sIgM- cells is shown. (TIFF) [file pgen.1005411.s008.tiff]

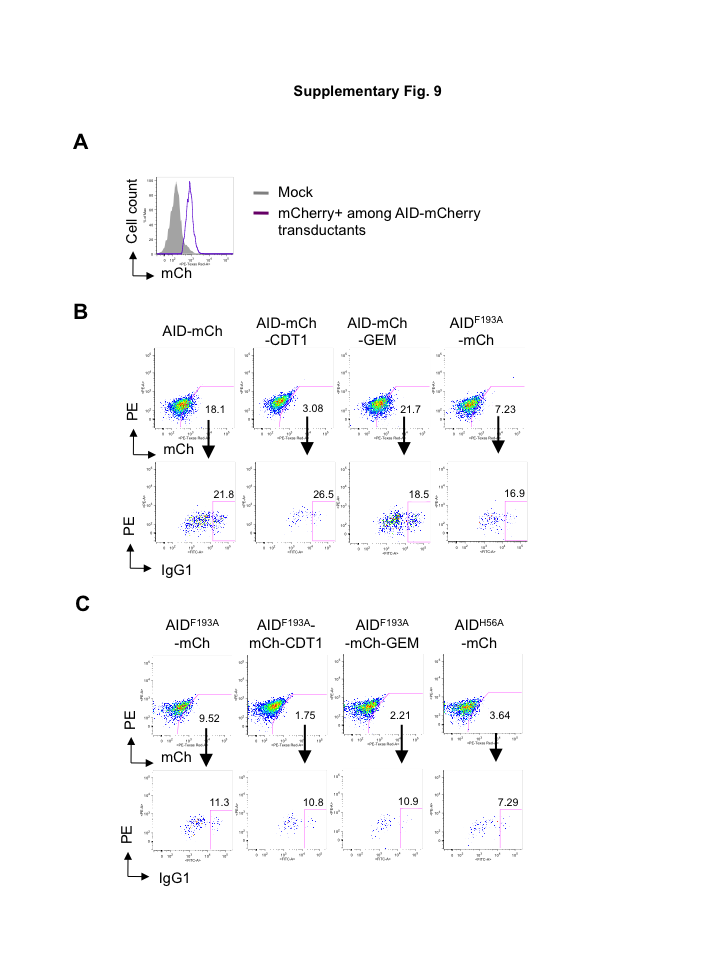

Supplement: S9 Fig — (A) Expression level of AID-mCherry transductants showing MFIs of mock transductants and mCherry+ cells among AID-mCherry transductants. (B) Flow cytometry of indicated transductants of primary murine splenic B cells, showing percent of cells that are mCherry+ (above) and fraction of IgG1+ cells among mCherry+ cells (below) at day 4 post transduction. (C) Flow cytometry of indicated transductants of primary murine splenic B cells, showing percent of cells that are mCherry+ (above) and fraction of IgG1+ cells among mCherry+ cells (below) at day 5 post transduction. (TIFF) [file pgen.1005411.s009.tiff]

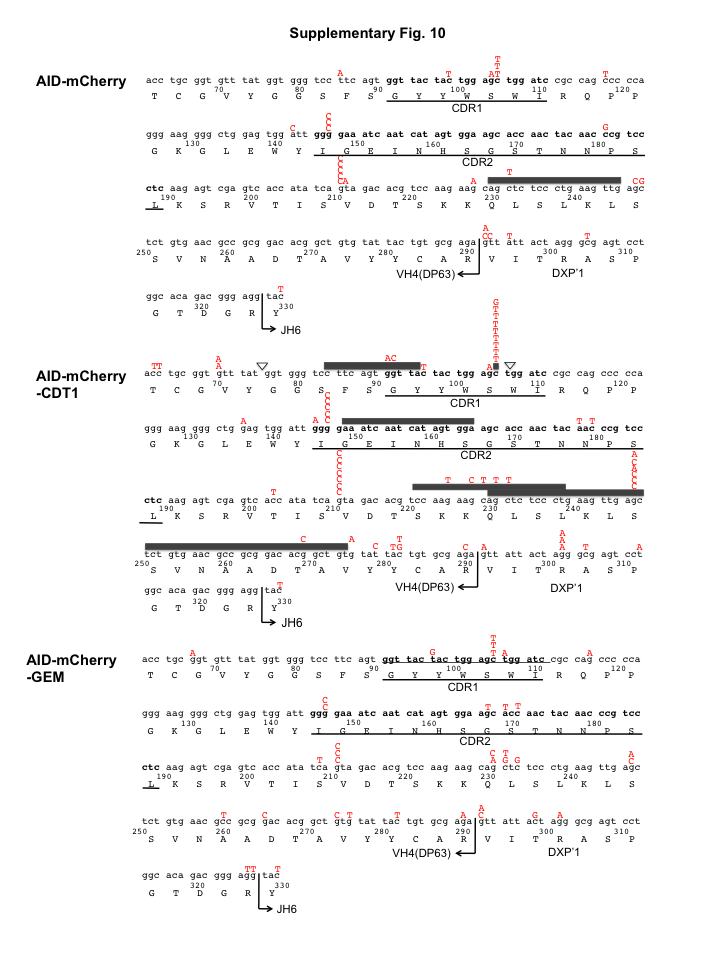

Supplement: S10 Fig — The parental nucleic acid sequence is shown in black, with positions of nucleotides numbered starting from the first base of first codon, corresponding amino acids are shown below each codon, and CDR1 and CDR2 underlined. Above the parental sequence, point mutations are shown in red, deletions as black bars and insertions as open triangles. Only sequences with unique mutation spectrum are shown. (TIFF) [file pgen.1005411.s010.tiff]

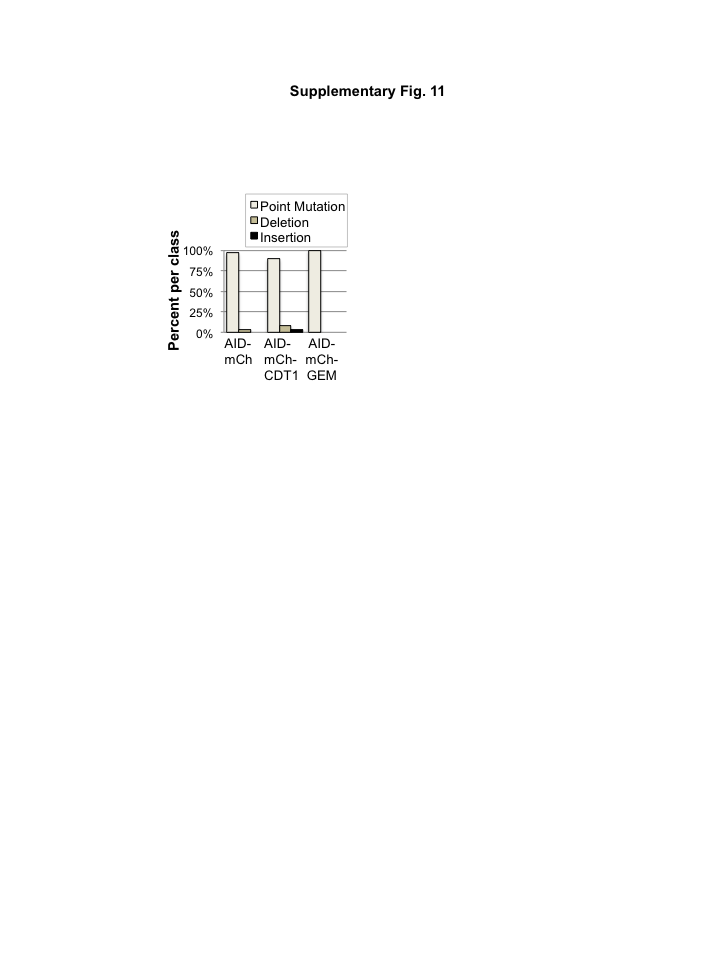

Supplement: S11 Fig — Percent of point mutations, deletions, and insertions in mutated VH regions of AID-mCherry, AID-mCherry-CDT1, or AID-mCherry-GEM transductants. (TIFF) [file pgen.1005411.s011.tiff]

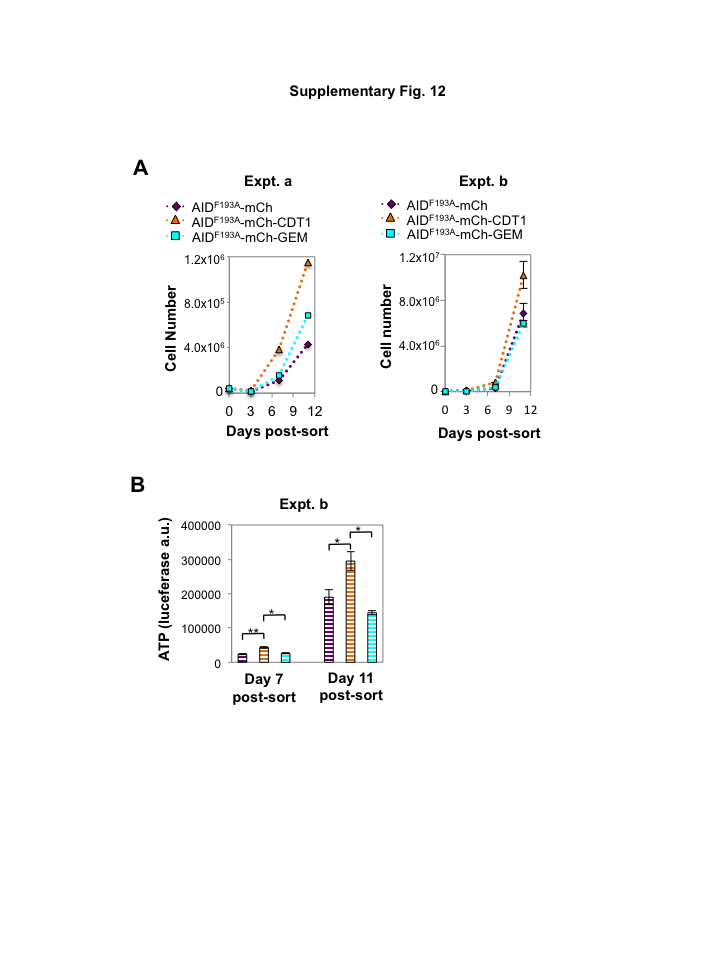

Supplement: S12 Fig — (A) Cell viability of indicated transductant populations, as determined by trypan blue exclusion. These independent populations were cultured at lower (Expt. a) and higher (Expt. b) density than the experiment shown in the text (Fig 5E), to ensure that cell density did not account for differences in relative viability. Viability was determined at the indicated day after sorting mCherry+ cells among recent transductants. (B) Cell viability of indicated transductant populations, as determined by assaying ATP levels at days 7 and 11 post-sorting mCherry+ cells among recent Ramos transductants. Viability of the population shown was also analyzed by trypan blue exclusion, and those in Expt. b in panel A, above. (TIFF) [file pgen.1005411.s012.tiff]

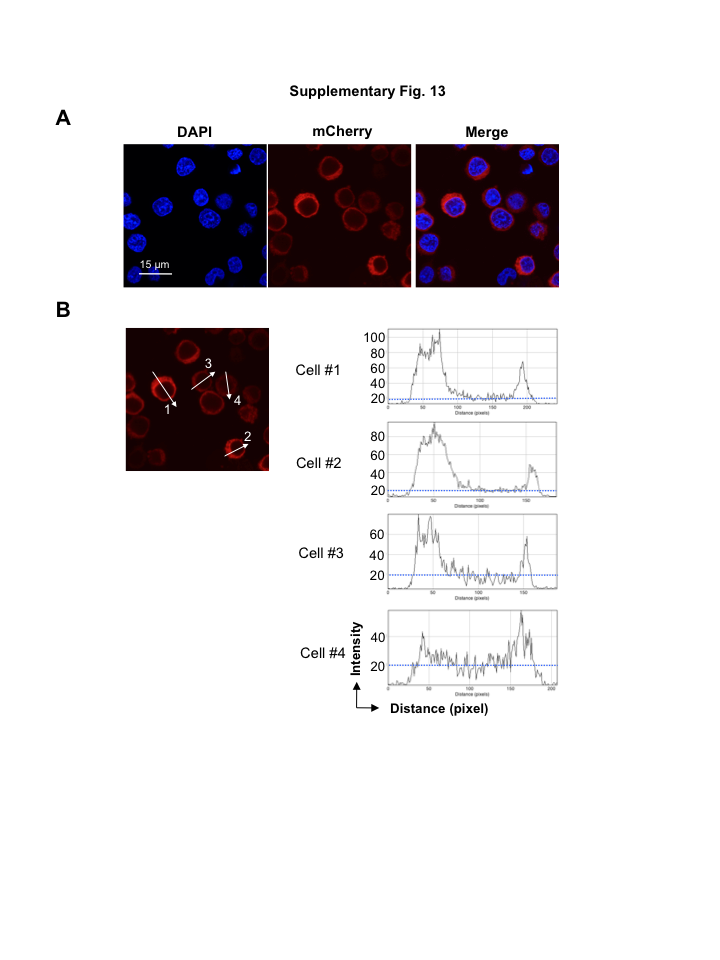

Supplement: S13 Fig — (A) Fluorescence images of AID-mCherry transductants acquired by confocal fluorescent microscopy. DAPI (left), mCherry (middle) and merge (right) signals are shown. (B) Representative individual AID-mCherry transductants (1–4 in image on left) and plot files of their mCherry fluorescence intensities along arbitary lines as indicated. Note the range of maximum fluorescence intensities. (TIFF) [file pgen.1005411.s013.tiff]

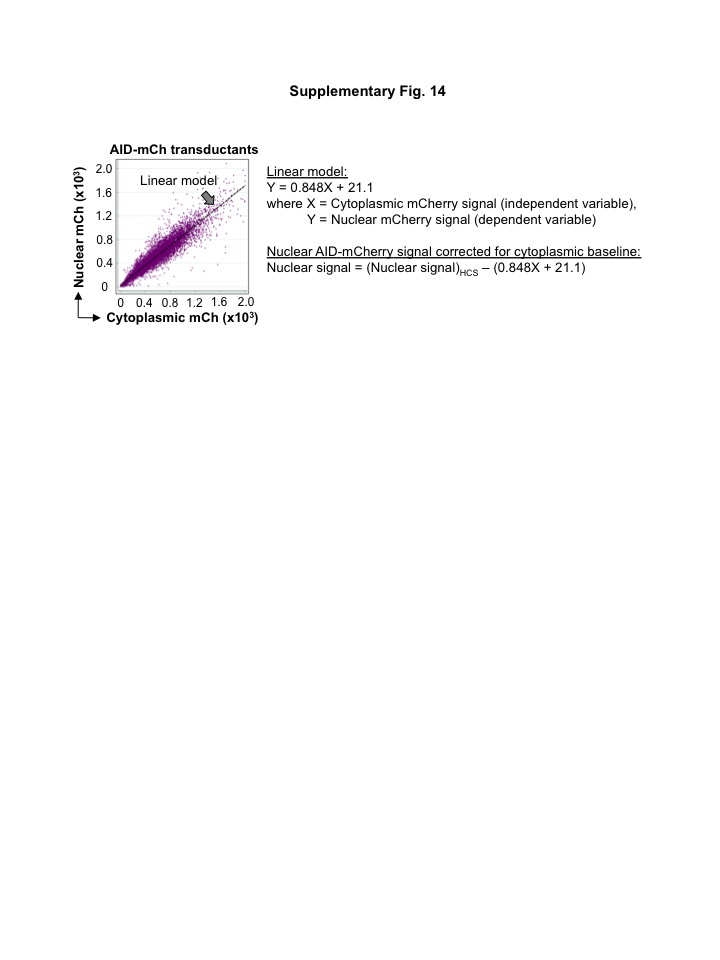

Supplement: S14 Fig — Scatter plot of nuclear vs. cytoplasmic mCherry signals of Ramos AID-mCherry transductants. Dashed line represents the linear model obtained from linear regression analysis. Right, the equation for the linear model is shown. Nuclear signals as determined by HCS were corrected for cytoplasmic baseline using the formula shown (see Materials and Methods). (TIFF) [file pgen.1005411.s014.tiff]

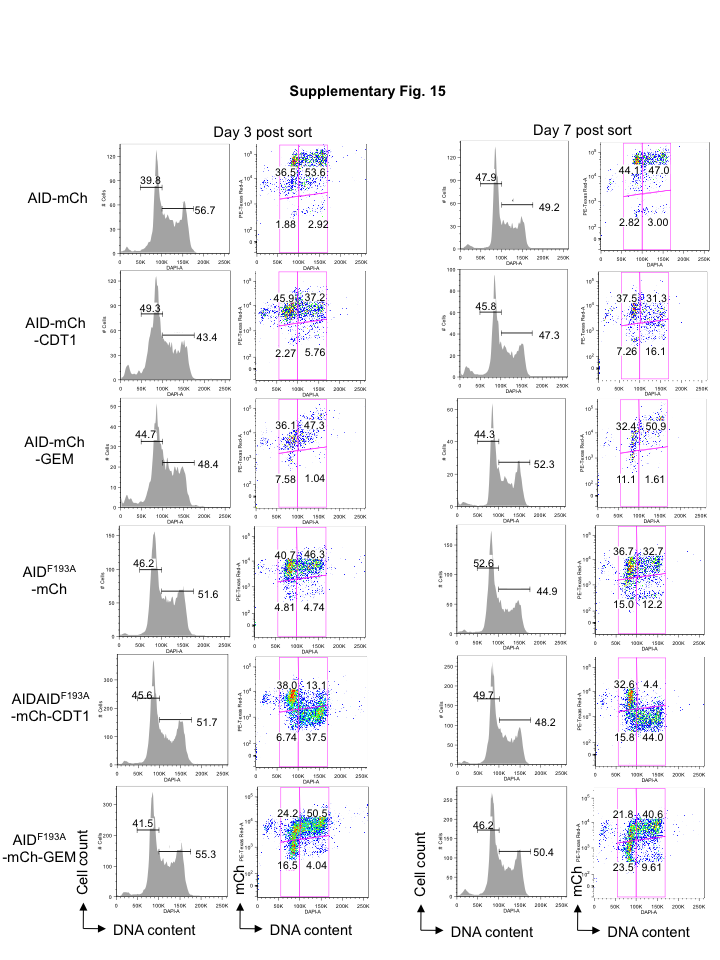

Supplement: S15 Fig — Flow cytometry of Ramos AID-mCherry, AID-mCherry-CDT1, AID-mCherry-GEM, AIDF193A-mCherry, AIDF193A-mCherry-CDT1 and AIDF193A-mCherry-GEM transductants, showing cell number relative to DNA content and percent of cells in G1 or S-G2/M phases (left), and mCherry signal and fraction of population in each quadrant (right) for day 3 and day 7 post sort. (TIFF) [file pgen.1005411.s015.tiff]
