# Supplementary material for: Cell Cycle Regulates Nuclear Stability of AID and Determines the Cellular Response to AID
Source: PLoS Genet. 2015 Sep 10;11(9):e1005411. doi: 10.1371/journal.pgen.1005411 (PMC4565580; doi:10.1371/journal.pgen.1005411)
Supplement: S1 Table — Statistical tests were performed using two-tailed, unpaired Student’s t-test, assuming unequal variances, for comparison of nuclear and cytoplasmic AID-mCherry signal and the N/C ratio between different treatment groups and between different times post treatment and untreated control in each treatment group. (DOCX) [file pgen.1005411.s016.docx]

**S1 Table. Probability tests for Fig. 1D.**

**AID-mCherry Transductants: Treated vs. Untreated**

**AID-mCherry Transductants: LMB vs. LMB + MG132 Treated**

**AID-mCherry Transductants: LMB vs. MG132 Treated**

**AID-mCherry Transductants: MG132 vs. LMB+MG132 Treated**
