# Supplementary material for: Cell Cycle Regulates Nuclear Stability of AID and Determines the Cellular Response to AID
Source: PLoS Genet. 2015 Sep 10;11(9):e1005411. doi: 10.1371/journal.pgen.1005411 (PMC4565580; doi:10.1371/journal.pgen.1005411)
Supplement: S2 Table — Statistical tests were performed using two-tailed, unpaired Student’s t-test, assuming unequal variances, for comparison of nuclear and cytoplasmic AID-mCherry signal and the N/C ratio between G1 and S; G1 and G2/M; and S and G2/M at different times post-treatment in each treatment group. (DOCX) [file pgen.1005411.s017.docx]

**S2 Table. Probability test for Fig. 2A: Cell Cycle Comparisons**

**S2 Table. Probability tests for Fig. 2A: Comparisons of Treated to Untreated Cells**
