# Supplementary material for: Cell Cycle Regulates Nuclear Stability of AID and Determines the Cellular Response to AID
Source: PLoS Genet. 2015 Sep 10;11(9):e1005411. doi: 10.1371/journal.pgen.1005411 (PMC4565580; doi:10.1371/journal.pgen.1005411)
Supplement: S3 Table — The number of cells (N) and the mean total, cytoplasmic, and nuclear mCherry signals are tabulated for Ramos AID-mCherry, AID-mCherry-CDT1, AID-mCherry-GEM, AIDF193A-mCherry, AIDF193A-mCherry-CDT1 and AIDF193A-mCherry-GEM transductants. Nuclear signals as determined by HCS were corrected for cytoplasmic baseline (see Materials and Methods). Statistical tests were performed using two-tailed, unpaired Student’s t-test, assuming unequal variances for comparisons among transductant populations. (DOCX) [file pgen.1005411.s018.docx]

**S3 Table. Subcellular Distribution of AID Determined by HCS Microscopy.**
